# Surface layer protein A from hypervirulent *Clostridioides difficile* ribotypes induce significant changes in the gene expression of tight junctions and inflammatory response in human intestinal epithelial cells

**DOI:** 10.1186/s12866-022-02665-0

**Published:** 2022-10-27

**Authors:** Maryam Noori, Masoumeh Azimirad, Gita Eslami, Mehdi Azizmohammad Looha, Abbas Yadegar, Zohreh Ghalavand, Mohammad Reza Zali

**Affiliations:** 1 Department of Microbiology, School of Medicine, Shahid Beheshti University of Medical Sciences, Tehran, Iran; 2 Foodborne and Waterborne Diseases Research Center, Research Institute for Gastroenterology and Liver Diseases, Shahid Beheshti University of Medical Sciences, Tehran, Iran; 3 Basic and Molecular Epidemiology of Gastrointestinal Disorders Research Center, Research Institute for Gastroenterology and Liver Diseases, Shahid Beheshti University of Medical Sciences, Tehran, Iran; 4 Gastroenterology and Liver Diseases Research Center, Research Institute for Gastroenterology and Liver Diseases, Shahid Beheshti University of Medical Sciences, Tehran, Iran

**Keywords:** *Clostridioides difficile*, SlpA, Tight junction, TLR-4, Inflammation, HT-29 cells

## Abstract

**Background:**

Surface layer protein A (SlpA), the primary outermost structure of *Clostridioides difficile*, plays an essential role in *C. difficile* pathogenesis, although its interaction with host intestinal cells are yet to be understood. The aim of this study was to investigate the effects of SlpA extracted from *C. difficile* on tight junction (TJ) proteins expression and induction of pro-inflammatory cytokines in human colon carcinoma cell line HT-29. SlpA was extracted from three toxigenic *C. difficile* clinical strains including RT126, RT001, RT084 as well as *C. difficile* ATCC 700057 as non-toxigenic strain. Cell viability was performed by MTT assay, and the mRNA expression of TJ proteins and inflammation-associated genes was determined using quantitative RT-PCR. Additionally, the secretion of IL-8, IL-1β and TNF-α cytokines was measured by ELISA.

**Results:**

*C. difficile* SlpA from selected RTs variably downregulated the expression level of TJs-assassinated genes and increased the expression level of TLR-4 and pro-inflammatory cytokines in HT-29 treated cells. SlpA from RT126 significantly (p_adj_<0.05) decreased the gene expression level of claudins family and JAM-A and increased the secretion of IL-8, TNF-α and IL1-β as compared to untreated cells. Moreover, only SlpA from RT001 could significantly induce the expression of IL-6 (p_adj_<0.05).

**Conclusion:**

The results of the present study highlighted the importance of SlpA in the pathogenesis of CDI and *C. difficile*-induced inflammatory response in the gut. Further studies are required to unravel the significance of the observed results in promoting the intestinal inflammation and immune response induced by *C. difficile* SlpA from different RTs.

**Supplementary Information:**

The online version contains supplementary material available at 10.1186/s12866-022-02665-0.

## Introduction

*Clostridioides difficile* (*C. difficile*), a Gram-positive, spore-forming, anaerobic bacterium, is the leading cause of antibiotic-associated diarrhea with substantial mortality [1]. *C. difficile* infection (CDI) can range from mild to acute watery diarrhea, pseudomembranous colitis, toxic megacolon and death [2]. CDI most commonly occurs in hospitalized elderly patients, however, in the past decade, community-acquired CDI has been increased particularly in newly affected population including young people and children [3–5].

The intestinal epithelial barrier is the primary basic defense against foodborne pathogens [6]. This layer is mainly composed of polarized intestinal epithelial cells (IECs), with distinct apical and basolateral surfaces sealed by tight junction (TJ) proteins [7]. The TJ complex is made up of several membrane and cytoplasmic proteins including claudins, occludins, junctional adhesion molecules (JAMs) and zonula occludens protein (ZO), that play pivotal roles in maintaining the integrity and homeostasis of the intestinal barrier [8]. Also, IECs contribute to developing the mucosal immune system in the intestine and can secrete a broad range of immunomodulatory cytokines and chemokines in response to potentially pathogenic bacteria [9]. However, some enteric bacterial pathogens such as *C. difficile* have been reported to disrupt the intestinal epithelial barrier, which may lead to intestinal inflammation by the secretion of pro-inflammatory cytokines from innate and acquired immune cells [10].

*C. difficile* strains produce two large potent toxins including cytotoxin A (TcdA) and enterotoxin B (TcdB) as the primary virulence determinants involved in the pathogenesis of CDI. These toxins cause marked detachment of IECs, TJ disruption and induction of inflammatory cytokines through activation of NF-κB, AP-1 and inflammasome [11–13]. In addition, surface layer proteins, known as the second class of *C. difficile* virulence attributes, have been shown to be involved in the colonization process and induction of pro-inflammatory response [14, 15]. Surface layer protein A (SlpA), the predominant outermost structure of the *C. difficile* surface layer, is composed of two distinct proteins, high molecular weight (HMW) and low molecular weight (LMW) subunits, which are derived from a single precursor (SlpA) encoded by *slpA* gene [15, 16]. The LMW-SlpA represents large sequence variability among different strains allowing bacteria to escape from the immune response, and consequently may enhance reinfection [17]. In previous in vitro [18] and in vivo [19] studies, the purified and recombinant SlpA subunits was demonstrated to cause significant reduction in *C. difficile* colonization. Similar to lipopolysaccharide (LPS), it has been well-documented that SlpA can also interact with toll-like receptor 4 (TLR-4), which results in induction of inflammatory responses [20].

While the function of *C. difficile* toxins has been largely described in the development of CDI, the role of its other virulence factors is poorly investigated. Here, we examined the effects of SlpA extracted from three toxigenic *C. difficile* clinical ribotypes (RT001, RT126, RT084) and *C. difficile* ATCC 700057 (RT038) as non-toxigenic strain on cell viability, gene expression of TJ proteins and inflammation-associated genes, and production of pro-inflammatory cytokines using human colon carcinoma cell line HT-29. Our study provides new insights into SlpA-mediated bacterial pathogenicity and broadens our knowledge of host-pathogen interactions in CDI.

## Materials and methods

### ***C. difficile *strains**

Three toxigenic *C. difficile* clinical strains belonging to different PCR RTs (RT001, RT126, RT084) and *C. difficile* ATCC 700057 (RT038) as non-toxigenic strain were used in this study (Table 1). These three clinical strains were selected due to their higher predominance in our previous study [21], and also according to the phylogenic analysis from our previous work [22]. Moreover, the amino acid sequences from these strains were compared and realigned with the SlpA sequences of known hypervirulent RTs (including RT027, RT078 and RT012 with the following accession numbers BAE79474, AAZ05994, AAZ05975, respectively) obtained from the GenBank/NCBI database (https://www.ncbi.nlm.nih.gov/genbank/) (Fig. S1).

**Table 1:** *C. difficile* strains used in the present study

Strain	PCR ribotype	SlpA type	Toxin profile
RIGLD-141	RT126	078 − 1	TcdA^+^/TcdB^+^/CdtA^ـــ^/CdtB^ـــ^
RIGLD-301	RT001	gr-1	TcdA^+^/TcdB^+^/CdtA^ـــ^/CdtB^ـــ^
RIGLD-309	RT084	cr-1	TcdA^+^/TcdB^ـــ^/CdtA^ـــ^/CdtB^ـــ^
ATCC 700057	RT038	ND	TcdA^ــــ^/TcdB^ـــ^/CdtA^ـــ^/CdtB^ـــ^

RT, ribotype; ND, not determined

### **Preparation of SlpA from* C. difficile***

Enriched SlpA fractions were prepared using the low pH glycine extraction as described previously by Calabi et al. [23] with the following modifications. Briefly, *C. difficile* strains were cultivated in brain heart Infusion (BHI) broth supplemented with *C. difficile* selective supplement (Oxoid) and 0.05% (w/v) L-cysteine (Sigma-Aldrich, USA) for 48–72 h at 37 °C under anaerobic conditions (85% N_2_, 10% CO_2_, and 5% H_2_). The strains were grown to exponential phase in TY medium and harvested by centrifugation (3000 g for 20 min). Pelleted cells were resuspended in 0.2 M glycine (Sigma-Aldrich, USA) pH 2.2 and incubated at room temperature with rotation for 30 min. After removal of the bacterial cell pellets by centrifugation (16,000 g for 15 min at 4 °C), the resultant SlpA-containing supernatants were collected, neutralized using 2 M Tris and stored for further analysis. The HMW and LMW subunits were separated by 12% SDS-PAGE gels stained with Coomassie brilliant blue. The concentration of purified SlpA proteins was determined by using a bicinchoninic acid (BCA) protein assay kit (Thermo Fisher Scientific, USA). The presence of LPS in the purified proteins was evaluated by LAL Chromogenic Endotoxin Quantitation Kit (Thermo Fisher Scientific, USA) according to the manufacturer’s instructions. Additionally, the existence of total TcdA and TcdB in the extracted SlpA proteins was determined by ELISA using the C. DIFFICILE TOX A/B II kit (TechLabs, Blacksburg, VA) following the manufacturer’s instructions. All determinations were tested in triplicate.

### Cell culture

The human HT-29 cell line was purchased from the Pasteur Institute, Tehran, Iran. The cells were grown in Dulbecco’s Modified Eagle Medium (DMEM) supplemented with 10% (v/v) heat-inactivated fetal bovine serum (Gibco/Invitrogen, USA), 2 mM of L-glutamine, 100 U/mL of penicillin, and 100 µg/mL of streptomycin, and were incubated in a 5% CO_2_ humidified atmosphere at 37 °C. The cells were cultured for 21 days to reach the full differentiation stage. The growth medium was refreshed every 2 days.

### Cell viability assay

Cell viability was measured by a quantitative colorimetric assay using the Cell Proliferation Kit I (Sigma-Aldrich, USA) following the manufacturer’s protocol. Briefly, 5 × 10^5^ cells/well were seeded in 96-well plates and allowed to adhere overnight. Cells were treated with varying concentrations of SlpA (15, 20, 25 µg/mL) at different time points (4, 8, 12, 24 and 48 h). After incubation period, 10 µL of 3-(4,5-dimethyl-2-thiazolyl)-2,5-diphenyl-tetrazolium bromide (MTT) solution was added to each well and incubation continued for a further 4 h in a 5% CO_2_ incubator at 37 °C. The reaction was terminated by adding color stop solution, dimethyl sulfoxide (DMSO), and the absorbance was recorded at 560 nm using a microplate reader (ELx808, BioTek Instruments, Winooski, Vermont, USA). The percentage of cell viability of treated cells was calculated using the following formula: Cell viability (%) = (X × 100%)/Y, where “X” is the absorbance of treated cells and “Y” the absorbance of untreated cells [24].

### Cell culture treatment

HT-29 cells were counted and seeded at a density of 2 × 10^5^ cells/well in 24-well plates and grown in a CO_2_ incubator for 24 h. Prior to treatment, the 80–90% confluent monolayers were washed three times with PBS (pH 7.2), and the media were replaced with antibiotic/serum-free complete DMEM overnight. Then, the cells were treated with SlpA at concentration of 20 µg/mL, and LPS from *Escherichia coli* 0111: B4 (Sigma-Aldrich, USA) at concentration of 100 ng/mL for different time points (4, 8, 12, 24 h) as the positive control. The untreated HT-29 cells were harvested as the negative control group. The experiments were performed in duplicate and repeated at least three times. The cell supernatants were collected at the indicated time periods and were utilized for measurement of IL-8, IL-1β and TNF-α cytokines.

### RNA extraction

Total RNA was extracted from HT-29 treated cells using RNeasy Plus Mini kit (Qiagen, Germany) following the manufacturer’s instructions. RNA concentration and purity were determined using NanoDrop spectrophotometer (ND-1000, Thermo Scientific, USA) by the A260/280 ratio and distinct bands of ribosomal RNA (rRNA) were visualized on 2% agarose gel electrophoresis. The RNA samples were frozen at − 80 °C until used for gene expression analysis.

### Quantitative real-time PCR

The extracted RNAs were transcribed into cDNA using a BioFACT™ RT-Kit (BIOFACT, South Korea) according to the manufacturer’s protocol. Amplified cDNAs were subjected to qRT-PCR using BioFACT™ 2X Real-Time PCR Master Mix (BIOFACT, South Korea) in SYBR Green chemistry. PCR amplifications were performed with the Rotor-Gene® Q (Qiagen, Germany) real-time PCR system using the primers sequences indicated in Table S1. Each sample was analyzed in duplicate and the results of fold change in mRNA expression were given relative to the control samples using the comparative Ct formula “2^−ΔΔCT^”, and the RNA input was normalized against the housekeeping gene β-actin.

### Cytokine measurements

Cytokine measurements were performed using commercial IL-8, IL-1β and TNF-α ELISA kits (ZellBio, Germany) in accordance with the manufacturer’s instructions, and analyzed with Bio-Plex Manager 6.1 software (Bio-Rad, USA). These assays were performed in duplicate.

### Statistical analysis

Descriptive statistics were carried out using mean ± standard deviation (SD) and frequency (percentage) for continuous and categorical data. The bar plot was used to indicate the differences in cell viability, gene expression and cytokine measurements between various treatments. The Dunn’s test, one-way and repeated measure analysis of variance (ANOVA) were used to determine the statistical significance between the groups. All *P*-values in multiple comparison were adjusted by Bonferroni method. The value of gene expressions was represented by time and treatment using heatmap. All analyses were perfomed by R (version 4.0.2) and SPSS (version 26.0). Adjusted *P*-values (padj) less than 0.05 were considered statistically significant; *P < 0.05, **P < 0.01, ***P < 0.001 and ****P < 0.0001.

## Results

### **Characterization of SlpA from* C. difficile***

The extracted SlpA fractions from different *C. difficile* RTs were separated by SDS-PAGE according to low pH glycine extraction method (Fig. S2). The SDS-PAGE revealed the presence of two visible distinctive protein bands at the expected molecular weights of 42-45 kDa and 32-36 kDa for the HMW-SlpA and LMW-SlpA, respectively. Interestingly, the purified SlpA from RT001 and RT084 and ATCC 700057 similarly migrated, whereas the size of the LMW-SlpA from RT126 was discernibly smaller (~ 32 kD) than the other RTs. Previous studies have also reported diverse size and antigenicity for the LMW-SlpA [18, 24].

### Effects of SlpA on HT-29 cell viability

**Fig. 1:**
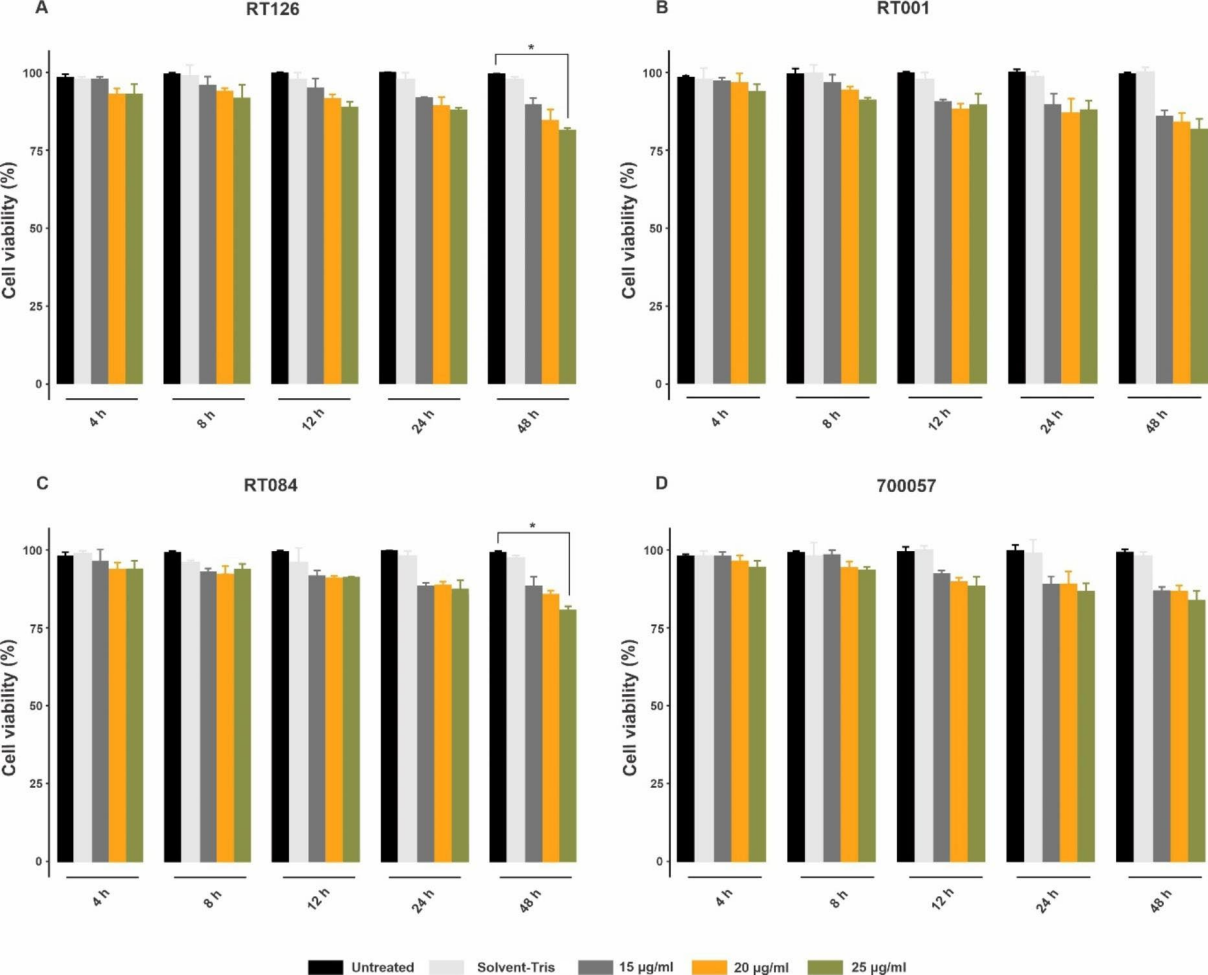
Cell viability determined by MTT assay. Different concentrations (15, 20, 25 µg/mL) of SlpA from (A) RT126 (B) RT001 (C) RT084 and (D) ATCC 700057 of *C. difficile* strains were added to HT-29 cells for different time points (4, 8, 12, 24 and 48 h) at 37 °C. Data were presented as mean ± SD from three independent experiments. Adjusted *P*-values (padj) less than 0.05 were considered statistically significant; *P < 0.05, **P < 0.01, ***P < 0.001 and ****P < 0.0001 by Bonferroni method and ANOVA statistical analysis. RT, ribotype

MTT assay was performed to evaluate the effects of SlpA on cell viability of HT-29 cells. As represented in Fig. 1, SlpA from different RTs and with various concentrations did not induce notable alterations in the number of viable cells at each study time point except for the concentration of 25 µg/mL of SlpA from RT084 (p_adj_0.016) and RT126 (p_adj_0.021) that significantly reduced the number of viable cells after 48 h of treatment. Thus, based on our results and previous reports [25, 26], the SlpA at concentration of 20 µg/mL was used for further experiments.

### **SlpA from select *C. difficile *strains reduce TJ gene expression in HT-29 cells**

**Fig. 2:**
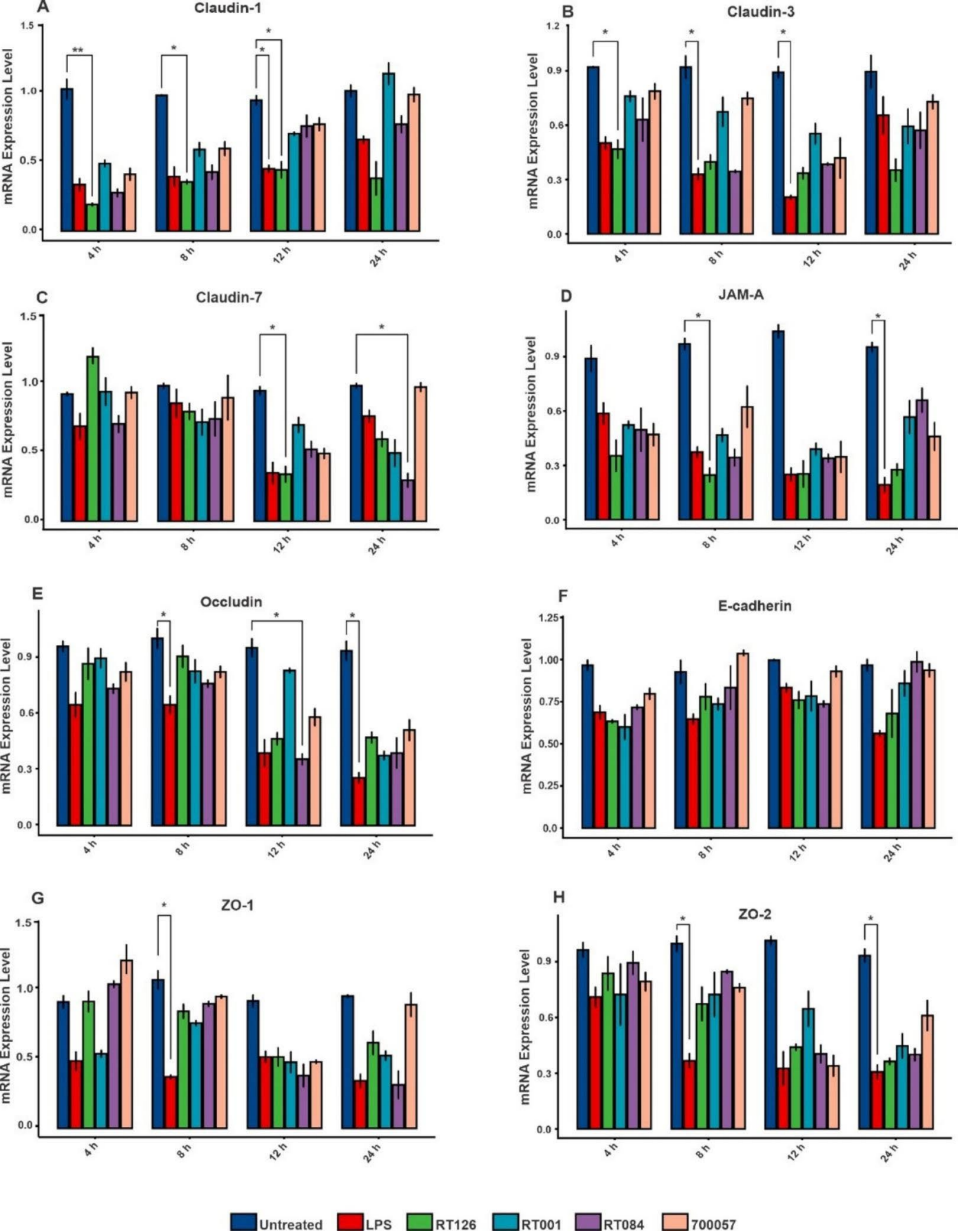
Relative mRNA levels of intestinal tight junction genes in HT-29 cells upon treatment with SlpA (20 µg/mL) from *C. difficile* (RT126, RT001, RT084) and *C. difficile* ATCC 700057 at different time points (4, 8, 12 and 24 h) measured by using quantitative real-time PCR assay. (A) Claudin-1, (B) Claudin-3, (C) Claudin-7, (D) JAM-A, (E) Occludin, (F) E-cadherin, (G) ZO-1, (H) ZO-2. Gene expression data were normalized to β-actin as the reference gene. Data were presented as mean ± SD from three independent experiments. Adjusted *P*-values (padj) less than 0.05 were considered statistically significant; *P < 0.05, **P < 0.01, ***P < 0.001 and ****P < 0.0001 by Bonferroni method and ANOVA statistical analysis. RT, ribotype

Disruption of the intestinal barrier is well-documented in CDI [27–29], whereby the bacteria penetrate in the mucus layer leading to elevated local and systemic inflammatory responses in the gut. To explore the effect of SlpA from different *C. difficile* RTs on the IECs barrier function and integrity, the gene expression of claudin-1, claudin-3, claudin-7, occludin, JAM-A, ZO-1, ZO-2 and E-cadherin were assessed upon treatment of HT-29 cells with extracted SlpA samples. Overall, the expression of TJs-associated genes was downregulated after exposure to SlpA from different RTs of *C. difficile*, in particular at 12 h post-infection, in comparison with untreated group (Fig. 2, Table S2). Accordingly, gene expression data obtained from heatmap analysis also displayed that the highest inhibitory effect of SlpA on expression of TJs-associated genes was occurred at 12 h post-infection (Fig. S3). In addition, the gene expression analysis showed that SlpA extracted from different RTs can variably downregulate the expression of TJs in HT-29 cells. As demonstrated in Fig. 2, SlpA from RT126 caused a significant decrease on gene expression level of claudin-1 at 4 h (p_Adj_0.008), 8 h (p_adj_0.047) and 12 h (p_adj_0.019), claudin-3 at 4 h (p_adj_0.032), claudin-7 at 12 h (p_adj_0.026), JAM-A at 8 h (p_adj_0.013) as compared to untreated cells. Also, a notable reduction in the gene expression of claudin-7 at 24 h (p_adj_0.037) and occludin at 12 h (p_adj_0.037) was found in treated cells with SlpA from RT084 in comparison with untreated cells. Moreover, SlpA of different RTs reduced the expression of E-cadherin, ZO-1 and ZO-2, although this reduction was not statistically significant (p_adj_>0.05) as compared with untreated control (Fig. 2 F-H).

### SlpA induces TLR-4 gene expression in HT-29 cells

**Fig. 3:**
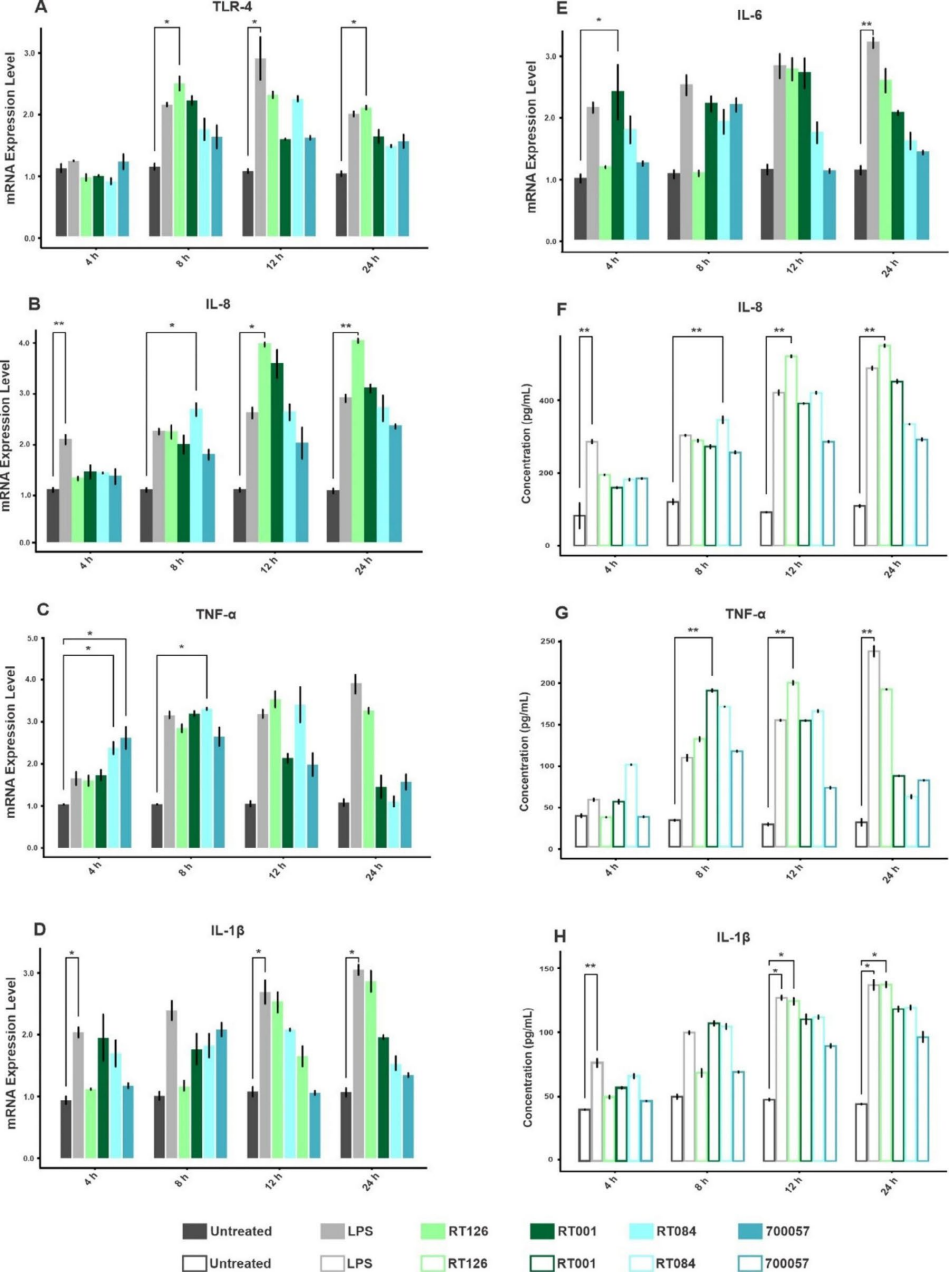
Relative mRNA levels and cytokine measurements. Relative expression of (A) TLR-4 (B) IL-8 (C) TNF-α (D) IL-1β, (E) IL-6 genes measured by using quantitative real-time PCR assay and inflammatory cytokines production of (F) IL-8, (G) TNF-α (H) IL-1β measured by ELISA in HT-29 cells upon treatment with SlpA (20 µg/mL) from *C. difficile* (RT126, RT001, RT084) and *C. difficile* ATCC 700057 at different time points (4, 8, 12 and 24 h). All Data were normalized to β-actin as the reference. Data were presented as mean ± SD from three independent experiments. Adjusted *P*-values (padj) less than 0.05 were considered statistically significant; *P < 0.05, **P < 0.01, ***P < 0.001 and ****P < 0.0001 by Bonferroni method and ANOVA statistical analysis. RT, ribotype

We sought to ascertain whether SlpA from different RTs of *C. difficile* can upregulate the TLR-4 gene expression in HT-29 cells. As shown in Fig. 3 A, SlpA from all different RTs increased the expression of TLR-4 in HT-29 cells when compared to the untreated cells. SlpA from RT126 induced a significant increase on gene expression of TLR-4 at 8 h (p_adj_0.011) and 24 h (p_adj_0.013) compared to the untreated control, while this upregulation was not statistically significant for other RTs (p_adj_>0.05). Besides, RT001 and RT084 induced the highest increase in the gene expression level of TLR-4 at 8 and 12 h, respectively, however this induction was not statistically significant.

### SlpA upregulates the gene expression of IL-8, TNF-α, IL-1β and IL-6 in HT-29 cells

Since SlpA is an immunodominant protein of *C. difficile* which interacts with IECs, we examined its impact on gene expression level of pro-inflammatory cytokines in HT-29 cells. As represented in Fig. 3 and Table S3, SlpA from almost all selected RTs induced pro-inflammatory responses in HT-29 cells, however SlpA from RT126 more typically enhanced the gene expression of inflammatory-related genes when compared to SlpA from other RTs. Moreover, SlpA from RT084 significantly induced IL-8 expression at 8 h (p_adj_0.011) (Fig. 3B), whilst SlpA from RT126 notably increased the mRNA expression level of IL-8 at 12 h (p_adj_0.009) and 24 h (p_adj_0.011) in comparison to the untreated cells. Interestingly, SlpA from our non-toxigenic ATCC 700057 strain caused a significant increase (p_adj_0.042) in gene expression of TNF-α at 4 h compered to untreated cells (Fig. 3 C). Also, SlpA from RT084 induced the gene expression of TNF-α at 4 h (p_adj_0.042) and 8 h (p_adj_0.019) post-infection. As shown in Fig. 3D, the gene expression of IL-1β was increased upon treatment of HT-29 cells with SlpA from different RTs, although in a non-significant relationship (p_adj_>0.05). Regarding the IL-6 expression (Fig. 3E), only SlpA from RT001 could significantly induce the expression of this cytokine at 4 h (p_adj_0.033) post-treatment in HT-29 cells.

### SlpA enhances the secretion of IL-8, TNF-α and IL-1β in HT-29 cells

ELISA was performed to further investigate the effect of SlpA on the production of IL-1β, IL-8 and TNF-α in HT-29 cells. As expected and shown in Fig. 3, SlpA from all different RTs increased the secretion of these pro-inflammatory cytokines from HT-29 cells. In more details, SlpA from RT084 significantly increased the production of IL-8 after 8 h of treatment (p_adj_0.034). In addition, SlpA from RT126 caused noticeable induction (p_adj_0.034) in the secretion of IL-8 from HT-29 cells after 12 and 24 h post-infection (Fig. 3 F). As demonstrated in Fig. 3G, SlpA extracted from RT001 and RT126 significantly enhanced the secretion of TNF-α in HT-29 cells after 8 (p_adj_0.034) and 12 h (p_adj_0.034) post treatment, respectively. The IL-1β was another pro-inflammatory cytokine that its production was increased in all treatment groups compared to untreated cells, however, the highest increase was induced by SlpA from RT126 after 12 and 24 h (p_adj_0.040) post-infection (Fig. 3 H). Overall, these results suggest that *C. difficile* SlpA protein, in particular SlpA purified from RT126, can stimulate the production of pro-inflammatory response in IECs.

## Discussion

In recent years, the increasing prevalence and severity of CDI as well as the emergence of new hypervirulent strains such as *C. difficile* RT027 and RT078, have become a critical concern for both public health and healthcare setting [30]. TcdA and TcdB, are the most studied virulence factors of *C. difficile* with UDP-glucosyltransferases properties that inactivate the Rho family of small GTPase. This inactivation of Rho GTPases consequently leads to disruption of TJs, induction of inflammatory cascades, a malabsorptive and secretory diarrhea, and tissue damage [12, 29, 31]. *C. difficile* SlpA is known as the second class of virulence attributes and an absolute necessity for CDI manifestation [14, 15].

**Fig. 4:**
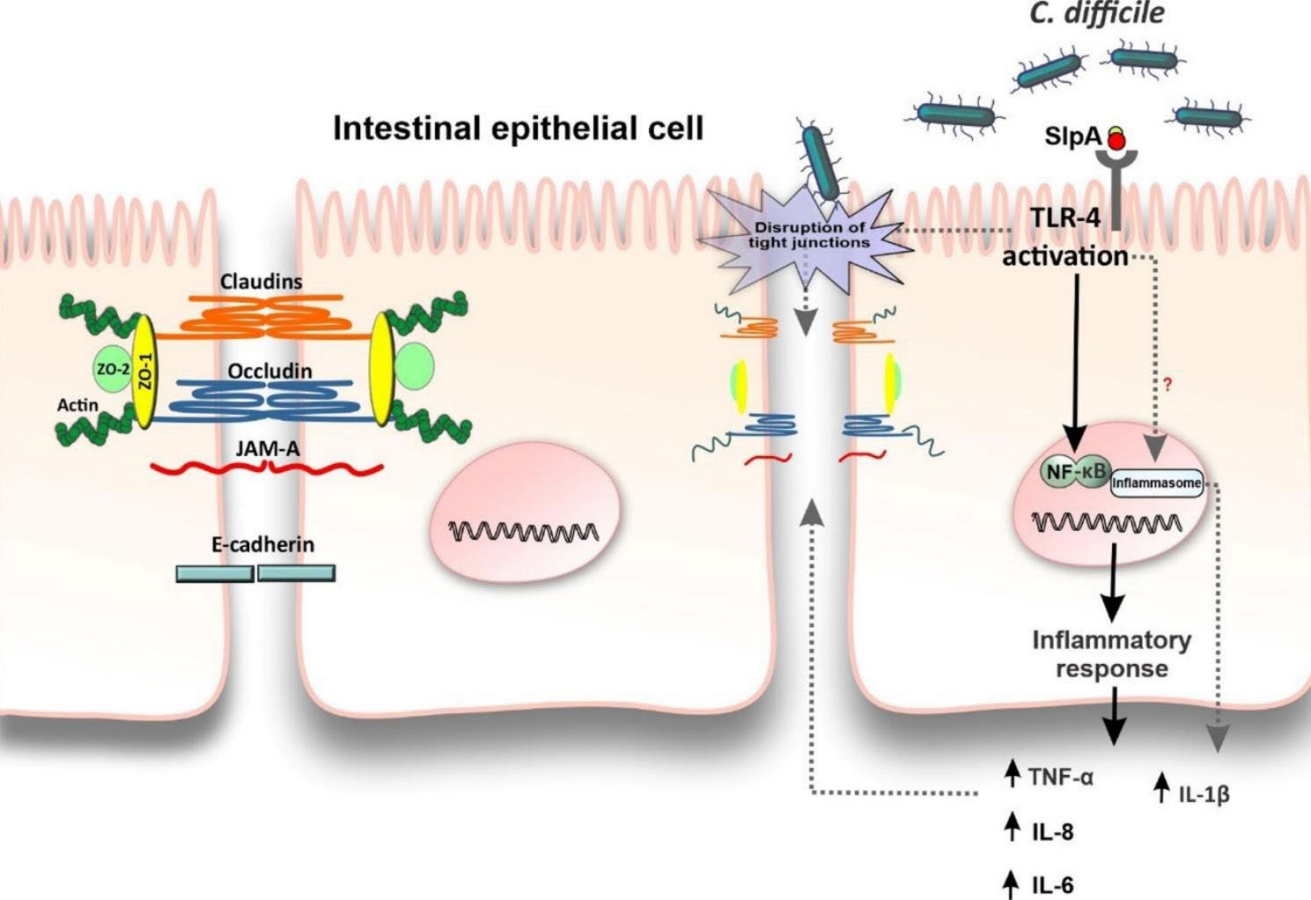
The effects of SlpA extracted from *C. difficile* clinical strains on human colon carcinoma cell line HT-29. SlpA binds to its receptor, TLR-4, results in increased expression and production of proinflammatory cytokines such as TNF-α, IL-6 and IL-8 in intestinal epithelial cells. SlpA from certain *C. difficile* RTs (SlpA purified from RT126) could significantly enhance IL-1β production in the gut, possibly through the bioprocessing of pro-IL-1β to its active form IL-1β following activation of the inflammasome pathway. Additionally, SlpA could distrupt tight junction and subsequently may increase intestinal permeability. RT, ribotype; TLR-4, toll-like receptor-4

So far, it has been explored in a number of studies that SlpA participates in the gut colonization process and adhesion to the intestinal surface mucosa [18]. Besides, SlpA may come into contact first with IECs, activate host cell bacterial recognition, promote inflammatory response and therefore possibly induce gut tissue damage. We previously found a great sequence diversity for the SlpA genotypes among clinical *C. difficile* strains in Iran [22]. In the present study, we demonstrated for the first time that *C. difficile* SlpA from different RTs including RT001, RT126, and RT084 are able to downregulate intestinal TJs-associated genes and upregulate the secretion of pro-inflammatory cytokines by IECs (Fig. 4).

It has been shown that *C. difficile* SlpA can be recognized by TLR-4 and induce both innate and adaptive immune responses by immune cells [20, 32]. We also found a significant upregulation in gene expression of TLR-4 in IECs upon treatment with purified *C. difficile* SlpA. Interestingly, SlpA from studied RTs could differentially induced TLR-4 gene expression, and the SlpA extracted from RT126 with toxin pattern of TcdA^+^/TcdB^+^ showed the highest impact on TLR-4 expression level. We previously reported RT126 as the most prevalent *C. difficile* RT among Iranian isolates [21, 22]. Additionally, based on the phylogenetic analysis presented in Fig. S1, we showed that SlpA from RT126 is closely related to RT078, a hypervirulent strain that causes severe disease in a younger population and is more frequently associated with community-acquired CDI [33]. Interestingly, in our recent 14-year-long cross-sectional study (2004 to 2018) we demonstrated that RT126 was mostly (69.2%) detected in adults [21]. Moreover, RT126 has a very similar ribotyping banding pattern to RT078 which is frequently reported in Asia, Europe and USA [34, 35].

Intestinal epithelial integrity defects can potentially lead to intestinal inflammation by allowing increased paracellular permeation and consequently systemic circulation of bacterial antigens. Several studies have demonstrated that LPS can cause downregulation in the expression of TJ-related genes, and also increase the intestinal paracellular permeability through activation of the TLR4-dependent pathway [36–39]. Our results showed that SlpA-treated cells may experience a trend of downregulation in the expression level of TJs-associated genes in comparison to untreated cells. In addition, we demonstrated that SlpA from toxigenic RT126 caused a notable downregulation on both JAM-A and claudin gene family (claudin-1, 3 and 7) when compared to control cells. The claudin family, known as the backbone of TJs, are crucial contributors to the maintenance of epithelial hemostasis [40]. Recently, Otani et al. proposed that claudins and JAM-A have an overlapping effect on regulating TJs function and epithelial polarity [41]. Moreover, Chen et al. reported profound disorganization of claudin-1, ZO-1, and occludin in IECs following TcdA exposure [42]. TcdA and TcdB were also reported to induce remarkable disruption of occludin, ZO-1, and ZO-2 in T84 colonic adenocarcinoma cell line [13]. Some pathogens, like *Clostridium botulinum* and *Helicobacter pylori*, have been found to disrupt homeostasis by cleaving E-cadherin [43], as a major component of adherens junctions, whereas *C. difficile* toxins were reported to have no influence on the partitioning of E-cadherin [13, 44]. In this study, we also did not find any significant alteration in the expression of E-cadherin in response to SlpA treatment. Our findings suggest that SlpA isolated from distinct *C. difficile* PCR RTs can differently induce epithelial barrier disruption of human IECs through downregulation of TJs and may provide *C. difficile* bacteria to successfully pass across the IECs monolayer. However, understanding the precise role of SlpA in the regulation of TJ proteins warrants further investigation.

Production of pro-inflammatory cytokines during mucosal inflammation can lead to epithelial TJ disruption and increased paracellular gut permeability [45–47]. In this study, we demonstrated that exposing IECs to SlpA from selected RTs of *C. difficile* induced considerable amounts of pro-inflammatory cytokines including IL-8, TNF-α, IL-1β and IL-6 in comparison with untreated cells. This noticeable inflammatory response may lead to the induction of intestinal tissue damage caused by *C. difficile*. In addition, our results showed that SlpA from different RTs elicited distinct immune responses in IECs. Among the measured cytokines, IL8 was the most abundant cytokine produced by *C. difficile* SlpA. In agreement with our findings, Vohra et al. revealed that the secretion of IL-8 was markedly elevated in response to *C. difficile* SLPs in macrophage cells [48]. Furthermore, we showed that SlpA from RT126 and RT084 induced more potent IL8 response in HT-29 cells relative to the SlpA from RT001. As mentioned earlier, SlpA from RT126 was closely associated with hypervirulent strain RT078. In addition, strains with RT084 were known as prototypic non-toxigenic strains and are reported to be prevalent in symptomatic patients [49, 50]. In contrast, the *C. difficile* RT084 strain that was used in this study merely produced toxin A (TcdA^+^). This finding once again highlights the significance of other virulence factors in the pathogenesis of various *C. difficile* strains. Besides, this profound induction of IL-8 by SlpA from RT126 and RT084 can be attributed to specific virulence features of their SLPs.

Our results also demonstrated that *C. difficile* SlpA from all selected RTs induced TNF-α secretion, and SlpA from RT126 and RT001 were able to induce TNF-α release at higher levels than other strains used in this work. Moreover, based on phylogenic analysis shown in Fig. S1, SlpA from RT001 was closely related to SlpA from hypervirulent strain RT027. Collin et al. showed that SlpA from RT001 could stimulate macrophage cells to produce elevated levels of TNF-α, IL-12p40, monocyte chemoattractant protein-1 (MCP-1) and macrophage inflammatory protein-1α (MIP-1α) in a similar manner to LPS [25]. TNF-α is a pro-inflammatory cytokine that plays a key role in driving systemic inflammatory response in CDI patients [51]. Besides, it has been demonstrated that both TcdA and TcdB toxins cause increased TNF-α gene expression in the colon of *C. difficile*-infected mice [52]. In addition, extensive research has documented that TNF-α is an important factor to change localization or expression of TJs, and subsequently disrupt intestinal epithelial barrier integrity [53–55].

IL-1β is produced as an inactive precursor called pro-IL-1β, which passes an autolytic cleavage by caspase1/inflammasome axis to form the biologically active form of IL-1β; this process is reported to happen through NLRP3 (NOD-, LRR- and pyrin domain-containing protein 3) inflammasome signaling pathway [56–58].In this study, we found that IECs stimulated with SlpA from RT126 could induce significant amount of IL-1β production as compared to untreated cells. It can be hypothesized that the increased secretion of IL-1β by SlpA from RT126 may be because of the plausible effect of this bacterial protein on the inflammasome activation and IL-1β bioprocessing in IECs (Fig. 4). Also, previous studies showed that SlpA from *C. difficile* RT001 failed to activate IRF3, as an indicator of inflammasome activation, while SlpA from RT027 activated both NFκB and IRF3 downstream of TLR-4 [20, 59]. Conversely, Collin et al. reported that SlpA from RT001 was able to induce significant production of IL-1β, IL-6 and MIP-2 even higher than LPS [25]. Moreover, TcdA and TcdB toxins have been shown to induce elevated secretion of IL-1β via the inflammasome activation [58, 60]. Jafari et al. also demonstrated that *C. difficile* strain 630Δerm *tcdA/tcdB* double-toxin mutant could not induce IL-1β secretion in bone-marrow-derived dendritic cells (BMDC), however both the single-toxin mutant strain and the parental toxigenic strain significantly induced the expression of IL-1β and NLRP3 [61]. Further investigations are needed to explore whether the mechanisms of inflammasome activation and IL-1β maturation in IECs correspond to those defined in macrophages.

Furthermore, there is strong evidence that IL-6 plays a crucial role in the inflammatory process in the gut [62]. In this work, we showed that SlpA from RT001 caused notable increase in gene expression of IL-6 in HT-29 cells in comparison to other RTs. Previous studies also exhibited that SlpA from RT001 could induce IL-6 production in immune cells including DCs and macrophages followed by DC maturation and activation of macrophage [20, 25]. Bianco et al. also found that SlpA from strains with RT001, RT012 and RT027 increased IL-6 production in monocytes and monocyte-derived dendritic cells (MDDCs) [63]. Also, it has been shown that IL-6 causes a drop in transcutaneous electrical resistance (TER) and increased TJ permeability in intestinal epithelial Caco-2 cells [64, 65]. These data suggest that the high level of inflammatory cytokines induced by SlpA might foster inflammation and tissue damage in the intestinal epithelium. However, recent data suggest that in vitro models are unable to fully mimic the physiologic aspects of the intestine and therefore further studies by using new systems like intestinal organoids are needed to meticulously unravel host-microbe interactions [66, 67].

## Conclusion

In conclusion, the results of the present study highlight the importance of SlpA in the pathogenesis of CDI and *C. difficile*-induced inflammatory response. Our data also demonstrate for the first time that *C. difficile* SlpA can trigger the disruption of the intestinal mucosal barrier. Furthermore, the purified SlpA proteins from the three selected *C. difficile* RTs, in particular SlpA purified from RT126, could variably downregulate the expression of intestinal TJs and induced the inflammatory-associated genes in vitro. Taken together, our findings also suggest that sequence diversity of SlpA within various *C. difficile* strains may influence the host-microbe interactions, and play a key role in driving the emergence of hypervirulent strains, and subsequently influence the pathology of CDI. Further studies, especially in vivo, are required to meticulously unravel the significance of the observed results in promoting the intestinal inflammation and immune response induced by *C. difficile* SlpA from different RTs.

## Electronic supplementary material

Below is the link to the electronic supplementary material.

Supplementary Material 1

Supplementary Material 2

Supplementary Material 3

Supplementary Material 4

Supplementary Material 5

Supplementary Material 6

## Data Availability

All data generated or analyzed during this study are included in this published article and its supplementary information.
