# Supplementary material for: Surface layer protein A from hypervirulent Clostridioides difficile ribotypes induce significant changes in the gene expression of tight junctions and inflammatory response in human intestinal epithelial cells
Source: BMC Microbiol. 2022 Oct 27;22:259. doi: 10.1186/s12866-022-02665-0 (PMC9608920; doi:10.1186/s12866-022-02665-0)
Supplement: Supplementary file 1 — Supplementary Material 1 [file 12866_2022_2665_MOESM1_ESM.docx]

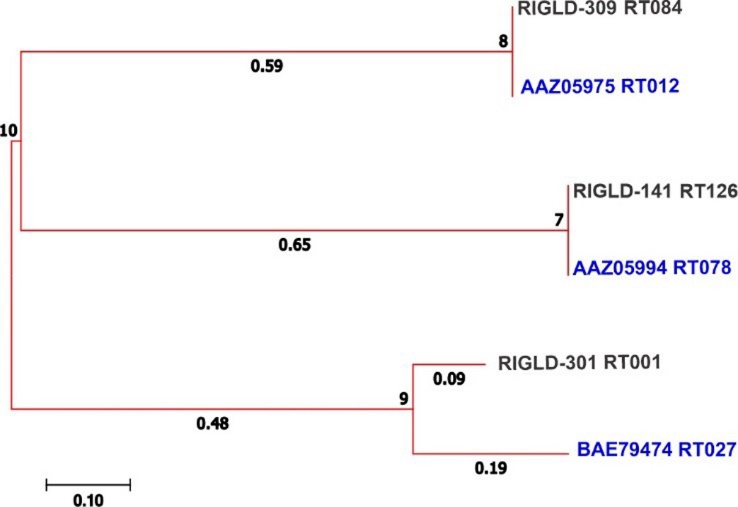


**Fig. S1** Phylogenetic analysis based on comparison between *slpA* sequences of *C. difficile* isolates from this study with *slpA* sequences deposited in public databases (determined by BLAST search and shown in blue). Maximum Likelihood tree using Poisson correction method was drown by MEGA 7.0 software with bootstrap method at 1000 replications. RT, ribotype
