# Supplementary material for: Surface layer protein A from hypervirulent Clostridioides difficile ribotypes induce significant changes in the gene expression of tight junctions and inflammatory response in human intestinal epithelial cells
Source: BMC Microbiol. 2022 Oct 27;22:259. doi: 10.1186/s12866-022-02665-0 (PMC9608920; doi:10.1186/s12866-022-02665-0)
Supplement: Supplementary file 2 — Supplementary Material 2 [file 12866_2022_2665_MOESM2_ESM.docx]

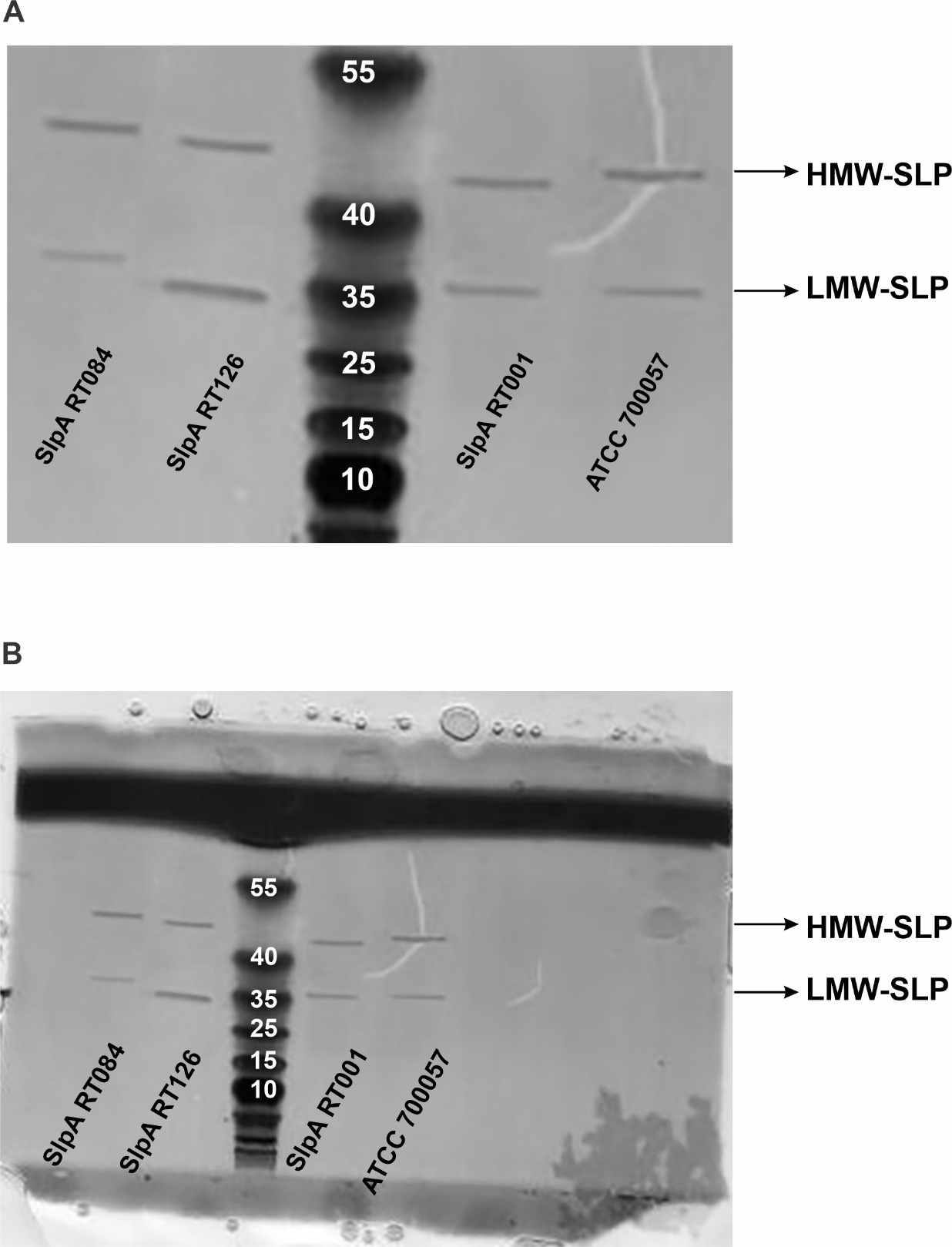


**Fig. S2** Cropped (A) and uncropped (B) blots of SDS-PAGE analysis of SlpA extracted from *C. difficile* (RT084, RT126, RT001) and *C. difficile* ATCC 700057 by using the low pH glycine extraction method. Molecular mass standards and their positions are indicated on the middle. RT, ribotype; HMW, high molecular weight; LMW, low molecular weight
