# Supplementary material for: Surface layer protein A from hypervirulent Clostridioides difficile ribotypes induce significant changes in the gene expression of tight junctions and inflammatory response in human intestinal epithelial cells
Source: BMC Microbiol. 2022 Oct 27;22:259. doi: 10.1186/s12866-022-02665-0 (PMC9608920; doi:10.1186/s12866-022-02665-0)
Supplement: Supplementary file 3 — Supplementary Material 3 [file 12866_2022_2665_MOESM3_ESM.docx]

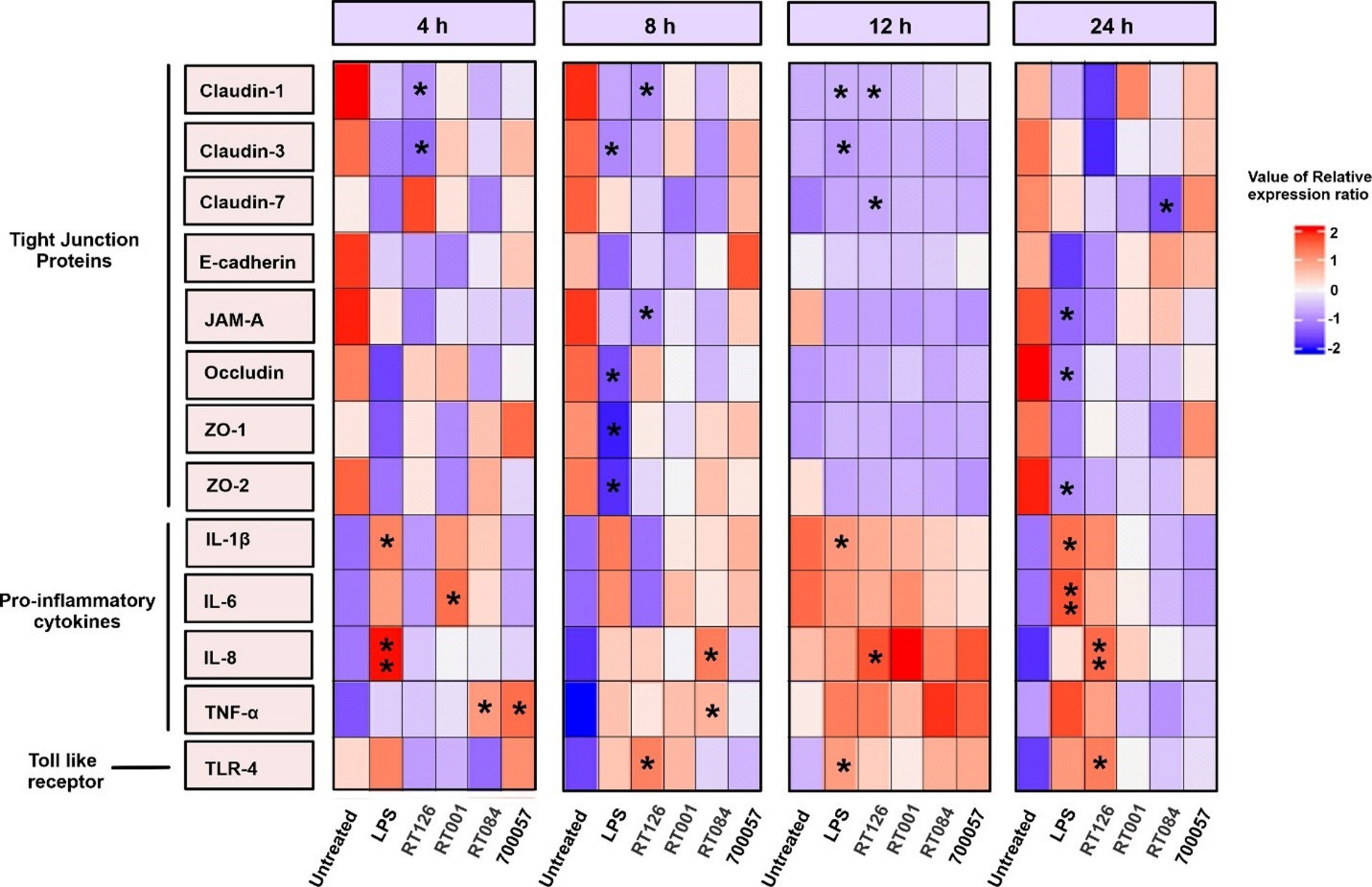


**Fig. S3** Gene expression heatmap of tight junction proteins and pro-inflammatory cytokines in HT-29 cells upon treatment with SlpA (20 µg/mL) from *C. difficile* (RT126, RT001, RT084) and *C. difficile* ATCC 700057 at different time points. Four boxes represent four time points in our experiment (4, 8, 12 and 24 h). In each box, columns correspond to the expression profile of treatments and each row presents the expression level of the gene whose name is indicated on the left. Value of relative expression ratio is indicated in the legend on the right. Boxes marked with asterisk shows statistically significant effect of treatment on gene expression, when p_adj_ was equal or less than 0.05. Heatmap was plotted using heatmap.2 function of the R/Bioconductor package gplots. RT, ribotype
