# Supplementary material for: Surface layer protein A from hypervirulent Clostridioides difficile ribotypes induce significant changes in the gene expression of tight junctions and inflammatory response in human intestinal epithelial cells
Source: BMC Microbiol. 2022 Oct 27;22:259. doi: 10.1186/s12866-022-02665-0 (PMC9608920; doi:10.1186/s12866-022-02665-0)
Supplement: Supplementary file 4 — Supplementary Material 4 [file 12866_2022_2665_MOESM4_ESM.docx]

**Table S1** Oligonucleotide sequences used in real-time PCR assays.

| Target gene | Oligonucleotide sequences (5′–3′) | Reference |
| --- | --- | --- |
| claudin-1 | F: TTTCCTGCTACAACAATCCTCTCC  R: GTTGTTTTTCGGGGACAGGAAC | [1] |
| claudin-3 | F: TGCTGTTCCTTCTCGCCGCC  R: CTTAGACGAAGTCCATGCGG | [2] |
| claudin-7 | F: CTGCAAAATGTACGACTCGGTG  R: GCAAGACCTGCCACGATGAAAA | [1] |
| occludin | F: CCCATCTGACTATGTGGAAAGA  R: AAACCGCTTGTCATTCACTTTG | [1] |
| E-cadherin | F: GTCAGTTCAGACTCCAGCCC  R: AAATTCACTCTGCCCAGGACG | [3] |
| JAM-A | F: GTGCCTTCAGCAACTCTTCC  R: GAGCCGATATCCGTTTGGTC | [4] |
| ZO-1 | F: GGGAACAACATACAGTGACGC  R: CCCCACTCTGAAAATGAGGA | [1] |
| ZO-2 | F: GGAGGATGTGCTTCATTCG  R: GGCCTCTTGACCACAATAG | [1] |
| TNF-α | F: CCCAGGGACCTCTCTCTAATC  R: ATGGGCTACAGGCTTGTCACT | [5] |
| Il-1β | F: AGGCTGCCGGGACTCACAGCA  R: TGAGGCCCAAGGCCACAGGT | [5] |
| IL-6 | F: GCACTGGCAGAAAACAACCT  R: TCAAACTCCAAAAGACCAGTGA | [5] |
| IL-8 | F: CTCTTGGCAGCCTTCCTGATT  R: ACTCTCAATCACTCTCAGTTCT | [5] |
| TLR-4 | F: CGAGGAAGAGAAGACACCAGT  R: CATCATCCTCACTGCTTCTGT | [6] |
| β-actin | F: CTGGAACGGTGAAGGTGACA  R: AAGGGACTTCCTGTAACAATGCA | [1] |

1. Duijghuijsen L, Grefte S, de Boer V, Zeper L, Dartel D, Stelt I, et al. Mitochondrial ATP Depletion Disrupts Caco-2 Monolayer Integrity and Internalizes Claudin 7. Front Physiol. 2017; 8(794), doi.org/10.3389/fphys.2017.00794.

2. Yamaguchi H, Kojima T, Ito T, Kimura Y, Imamura M, Son S, et al. Transcriptional Control of Tight Junction Proteins via a Protein Kinase C Signal Pathway in Human Telomerase Reverse Transcriptase-Transfected Human Pancreatic Duct Epithelial Cells. Am J Pathol. 2010; 177(2): 698-712, doi: 10.2353/ajpath.2010.091226.

3. Han YH, Kee JY, Hong SH. Rosmarinic Acid Activates AMPK to Inhibit Metastasis of Colorectal Cancer. Front Pharmacol. 2018; 9(68), doi.org/10.3389/fphar.2018.00068.

4. Wang K, Dong S, Higazy D, Jin L, Zou Q, Chen H, et al. Inflammatory Environment Promotes the Adhesion of Tumor Cells to Brain Microvascular Endothelial Cells. Front. Oncol, 2021; 11(2329), doi.org/10.3389/fonc.2021.691771

5. Shan X, Zhang Y, Chen H, Dong L, Wu B, Xu T,et al. Inhibition of epidermal growth factor receptor attenuates LPS-induced inflammation and acute lung injury in rats. Oncotarget, 2017; 8(16), [doi.org/10.18632/oncotarget.15790](https://doi.org/10.18632/oncotarget.15790)

6. Huang Y, Cai B, Xu M, Qiu Z, Tao Y, Zhang Y,et al. Gene Silencing of Toll-Like Receptor 2 Inhibits Proliferation of Human Liver Cancer Cells and Secretion of Inflammatory Cytokines. PLOS ONE, 2012; **7**(7): e38890, doi.org/10.1371/journal.pone.0038890
