# Supplementary material for: Surface layer protein A from hypervirulent Clostridioides difficile ribotypes induce significant changes in the gene expression of tight junctions and inflammatory response in human intestinal epithelial cells
Source: BMC Microbiol. 2022 Oct 27;22:259. doi: 10.1186/s12866-022-02665-0 (PMC9608920; doi:10.1186/s12866-022-02665-0)
Supplement: Supplementary file 5 — Supplementary Material 5 [file 12866_2022_2665_MOESM5_ESM.docx]

**Table S2** The multiple comparison of tight junction proteins and inflammation-associated genes including (A) claudin-1, (B) claudin-3, (C) claudin-7, (D) E-cadherin, (E) JAM-A, (F) occludin, (G) ZO-1, (H) ZO-2, (I) IL-1β, (J) IL-6, (K) IL-8, (L) TLR-4 and (M) TNF-α in HT-29 cells upon treatment with SlpA (20 µg/mL) from *C. difficile* (RT126, RT001, RT084) and *C. difficile* ATCC 700057 at different time points (4, 8, 12 and 24 h) measured by using quantitative real-time PCR assay.

**(A)**

| **Hour** | **Contrast** | **Test Statistic** | **Std. Error** | **Std. Test Statistic** | ***P*-value** | **Adj *P*-value^a^** |
| --- | --- | --- | --- | --- | --- | --- |
| **4 h** | RT126-RT084 | -4.00 | 4.35 | -0.92 | 0.358 | 1.000 |
|  | RT126-LPS | 5.50 | 4.35 | 1.27 | 0.206 | 1.000 |
|  | RT126-700057 | -8.83 | 4.35 | -2.03 | 0.042 | 0.633 |
|  | RT126-RT001 | -11.67 | 4.35 | -2.68 | 0.007 | 0.109 |
|  | RT126-Un | 15.00 | 4.35 | 3.45 | 0.001 | 0.008 |
|  | RT084-LPS | 1.50 | 4.35 | 0.35 | 0.730 | 1.000 |
|  | RT084-700057 | -4.83 | 4.35 | -1.11 | 0.266 | 1.000 |
|  | RT084-RT001 | 7.67 | 4.35 | 1.76 | 0.078 | 1.000 |
|  | RT084-Un | 11.00 | 4.35 | 2.53 | 0.011 | 0.171 |
|  | LPS-700057 | -3.33 | 4.35 | -0.77 | 0.443 | 1.000 |
|  | LPS-RT001 | -6.17 | 4.35 | -1.42 | 0.156 | 1.000 |
|  | LPS-Un | 9.50 | 4.35 | 2.19 | 0.029 | 0.433 |
|  | 700057-RT001 | 2.83 | 4.35 | 0.65 | 0.515 | 1.000 |
|  | 700057-Un | 6.17 | 4.35 | 1.42 | 0.156 | 1.000 |
|  | RT001-Un | 3.33 | 4.35 | 0.77 | 0.443 | 1.000 |
| **8 h** | RT126-RT084 | -2.83 | 4.34 | -0.65 | 0.514 | 1.000 |
|  | RT126-LPS | 0.33 | 4.34 | 0.08 | 0.939 | 1.000 |
|  | RT126-700057 | -8.50 | 4.34 | -1.96 | 0.050 | 0.753 |
|  | RT126-RT001 | -7.50 | 4.34 | -1.73 | 0.084 | 1.000 |
|  | RT126-Un | 12.83 | 4.34 | 2.96 | 0.003 | 0.047 |
|  | RT084-700057 | -5.67 | 4.34 | -1.31 | 0.192 | 1.000 |
|  | RT084-RT001 | 4.67 | 4.34 | 1.08 | 0.282 | 1.000 |
|  | RT084-Un | 10.00 | 4.34 | 2.30 | 0.021 | 0.319 |
|  | LPS-700057 | -8.17 | 4.34 | -1.88 | 0.060 | 0.899 |
|  | LPS-RT001 | -7.17 | 4.34 | -1.65 | 0.099 | 1.000 |
|  | LPS-Un | 12.50 | 4.34 | 2.88 | 0.004 | 0.060 |
|  | 700057-Un | 4.33 | 4.34 | 1.00 | 0.318 | 1.000 |
|  | RT001-Un | 5.33 | 4.34 | 1.23 | 0.219 | 1.000 |
|  | LPS-RT084 | -2.50 | 4.34 | -0.58 | 0.565 | 1.000 |
|  | RT001-700057 | -1.00 | 4.34 | -0.23 | 0.818 | 1.000 |
| **12 h** | RT126-RT084 | -8.50 | 4.35 | -1.95 | 0.051 | 0.762 |
|  | RT126-LPS | 1.00 | 4.35 | 0.23 | 0.818 | 1.000 |
|  | RT126-700057 | -8.50 | 4.35 | -1.95 | 0.051 | 0.762 |
|  | RT126-RT001 | -7.00 | 4.35 | -1.61 | 0.108 | 1.000 |
|  | RT126-Un | 14.00 | 4.35 | 3.22 | 0.001 | 0.019 |
|  | RT084-700057 | 0.00 | 4.35 | 0.00 | 1.000 | 1.000 |
|  | RT084-Un | 5.50 | 4.35 | 1.26 | 0.206 | 1.000 |
|  | LPS-700057 | -7.50 | 4.35 | -1.72 | 0.085 | 1.000 |
|  | LPS-RT001 | -6.00 | 4.35 | -1.38 | 0.168 | 1.000 |
|  | LPS-Un | 13.00 | 4.35 | 2.99 | 0.003 | 0.042 |
|  | 700057-Un | 5.50 | 4.35 | 1.26 | 0.206 | 1.000 |
|  | RT001-Un | 7.00 | 4.35 | 1.61 | 0.108 | 1.000 |
|  | LPS-RT084 | -7.50 | 4.35 | -1.72 | 0.085 | 1.000 |
|  | RT001-700057 | -1.50 | 4.35 | -0.34 | 0.730 | 1.000 |
|  | RT001-RT084 | -1.50 | 4.35 | -0.34 | 0.730 | 1.000 |
| **24 h** | RT126-RT084 | -5.83 | 4.36 | -1.34 | 0.181 | 1.000 |
|  | RT126-LPS | 2.67 | 4.36 | 0.61 | 0.540 | 1.000 |
|  | RT126-700057 | -9.83 | 4.36 | -2.26 | 0.024 | 0.360 |
|  | RT126-RT001 | -14.17 | 4.36 | -3.25 | 0.001 | 0.057 |
|  | RT126-Un | 11.50 | 4.36 | 2.64 | 0.008 | 0.124 |
|  | RT084-700057 | -4.00 | 4.36 | -0.92 | 0.359 | 1.000 |
|  | RT084-RT001 | 8.33 | 4.36 | 1.91 | 0.056 | 0.837 |
|  | RT084-Un | 5.67 | 4.36 | 1.30 | 0.193 | 1.000 |
|  | LPS-700057 | -7.17 | 4.36 | -1.64 | 0.100 | 1.000 |
|  | LPS-RT001 | -11.50 | 4.36 | -2.64 | 0.008 | 0.124 |
|  | LPS-Un | 8.83 | 4.36 | 2.03 | 0.043 | 0.639 |
|  | 700057-RT001 | 4.33 | 4.36 | 0.99 | 0.320 | 1.000 |
|  | 700057-Un | 1.67 | 4.36 | 0.38 | 0.702 | 1.000 |
|  | LPS-RT084 | -3.17 | 4.36 | -0.73 | 0.467 | 1.000 |
|  | Un-RT001 | -2.67 | 4.36 | -0.61 | 0.540 | 1.000 |

**(B)**

| **Hour** | **Contrast** | **Test Statistic** | **Std. Error** | **Std. Test Statistic** | ***P*-value** | **Adj *P*-value^a^** |
| --- | --- | --- | --- | --- | --- | --- |
| **4 h** | RT126-LPS | 1.50 | 4.34 | 0.35 | 0.730 | 1.000 |
|  | RT126-RT084 | -4.17 | 4.34 | -0.96 | 0.337 | 1.000 |
|  | RT126-RT001 | -7.33 | 4.34 | -1.69 | 0.091 | 1.000 |
|  | RT126-700057 | -8.67 | 4.34 | -2.00 | 0.046 | 0.690 |
|  | RT126-Un | 13.33 | 4.34 | 3.07 | 0.002 | 0.032 |
|  | LPS-RT084 | -2.67 | 4.34 | -0.61 | 0.539 | 1.000 |
|  | LPS-RT001 | -5.83 | 4.34 | -1.34 | 0.179 | 1.000 |
|  | LPS-700057 | -7.17 | 4.34 | -1.65 | 0.099 | 1.000 |
|  | LPS-Un | 11.83 | 4.34 | 2.72 | 0.006 | 0.097 |
|  | RT084-RT001 | 3.17 | 4.34 | 0.73 | 0.466 | 1.000 |
|  | RT084-700057 | -4.50 | 4.34 | -1.04 | 0.300 | 1.000 |
|  | RT084-Un | 9.17 | 4.34 | 2.11 | 0.035 | 0.522 |
|  | RT001-700057 | -1.33 | 4.34 | -0.31 | 0.759 | 1.000 |
|  | RT001-Un | 6.00 | 4.34 | 1.38 | 0.167 | 1.000 |
|  | 700057-Un | 4.67 | 4.34 | 1.07 | 0.283 | 1.000 |
| **8 h** | RT126-RT001 | -5.33 | 4.35 | -1.23 | 0.220 | 1.000 |
|  | RT126-700057 | -6.67 | 4.35 | -1.53 | 0.125 | 1.000 |
|  | RT126-Un | 10.00 | 4.35 | 2.30 | 0.021 | 0.322 |
|  | LPS-RT084 | -1.67 | 4.35 | -0.38 | 0.701 | 1.000 |
|  | LPS-RT001 | -8.67 | 4.35 | -1.99 | 0.046 | 0.693 |
|  | LPS-700057 | -10.00 | 4.35 | -2.30 | 0.021 | 0.322 |
|  | LPS-Un | 13.33 | 4.35 | 3.07 | 0.002 | 0.032 |
|  | RT084-RT001 | 7.00 | 4.35 | 1.61 | 0.107 | 1.000 |
|  | RT084-700057 | -8.33 | 4.35 | -1.92 | 0.055 | 0.829 |
|  | RT084-Un | 11.67 | 4.35 | 2.68 | 0.007 | 0.109 |
|  | RT001-700057 | -1.33 | 4.35 | -0.31 | 0.759 | 1.000 |
|  | RT001-Un | 4.67 | 4.35 | 1.07 | 0.283 | 1.000 |
|  | 700057-Un | 3.33 | 4.35 | 0.77 | 0.443 | 1.000 |
|  | LPS-RT126 | -3.33 | 4.35 | -0.77 | 0.443 | 1.000 |
|  | RT084-RT126 | 1.67 | 4.35 | 0.38 | 0.701 | 1.000 |
| **12 h** | RT126-RT084 | -1.33 | 4.35 | -0.31 | 0.759 | 1.000 |
|  | RT126-RT001 | -6.50 | 4.35 | -1.50 | 0.135 | 1.000 |
|  | RT126-700057 | -2.50 | 4.35 | -0.58 | 0.565 | 1.000 |
|  | RT126-Un | 10.17 | 4.35 | 2.34 | 0.019 | 0.290 |
|  | LPS-RT084 | -5.83 | 4.35 | -1.34 | 0.179 | 1.000 |
|  | LPS-RT001 | -11.00 | 4.35 | -2.53 | 0.011 | 0.170 |
|  | LPS-700057 | -7.00 | 4.35 | -1.61 | 0.107 | 1.000 |
|  | LPS-Un | 14.67 | 4.35 | 3.38 | 0.001 | 0.011 |
|  | RT084-RT001 | 5.17 | 4.35 | 1.19 | 0.234 | 1.000 |
|  | RT084-700057 | -1.17 | 4.35 | -0.27 | 0.788 | 1.000 |
|  | RT084-Un | 8.83 | 4.35 | 2.03 | 0.042 | 0.631 |
|  | RT001-Un | 3.67 | 4.35 | 0.84 | 0.399 | 1.000 |
|  | 700057-Un | 7.67 | 4.35 | 1.76 | 0.078 | 1.000 |
|  | LPS-RT126 | -4.50 | 4.35 | -1.04 | 0.300 | 1.000 |
|  | 700057-RT001 | 4.00 | 4.35 | 0.92 | 0.357 | 1.000 |

The multiple comparisons were not presented (the null hypothesis was retained) for time 24 h and therefore asymptotic significance is displayed.

**(C)**

| **Hour** | **Contrast** | **Test Statistic** | **Std. Error** | **Std. Test Statistic** | ***P*-value** | **Adj *P*-value^a^** |
| --- | --- | --- | --- | --- | --- | --- |
| **4 h** | RT084-LPS | 0.33 | 4.36 | 0.08 | 0.939 | 1.000 |
|  | RT084-700057 | -6.83 | 4.36 | -1.57 | 0.117 | 1.000 |
|  | RT084-RT001 | 7.00 | 4.36 | 1.61 | 0.108 | 1.000 |
|  | RT084-Un | 7.50 | 4.36 | 1.72 | 0.085 | 1.000 |
|  | RT084-RT126 | 13.33 | 4.36 | 3.06 | 0.002 | 0.053 |
|  | LPS-700057 | -6.50 | 4.36 | -1.49 | 0.136 | 1.000 |
|  | LPS-RT001 | -6.67 | 4.36 | -1.53 | 0.126 | 1.000 |
|  | LPS-Un | 7.17 | 4.36 | 1.64 | 0.100 | 1.000 |
|  | LPS-RT126 | -13.00 | 4.36 | -2.88 | 0.098 | 0.055 |
|  | 700057-RT001 | 0.17 | 4.36 | 0.04 | 0.969 | 1.000 |
|  | 700057-Un | 0.67 | 4.36 | 0.15 | 0.878 | 1.000 |
|  | 700057-RT126 | 6.50 | 4.36 | 1.49 | 0.136 | 1.000 |
|  | RT001-Un | 0.50 | 4.36 | 0.11 | 0.909 | 1.000 |
|  | RT001-RT126 | 6.33 | 4.36 | 1.45 | 0.146 | 1.000 |
|  | Un-RT126 | -5.83 | 4.36 | -1.34 | 0.181 | 1.000 |
| **12 h** | RT084-RT001 | 4.67 | 4.36 | 1.07 | 0.284 | 1.000 |
|  | RT084-Un | 7.67 | 4.36 | 1.76 | 0.079 | 1.000 |
|  | LPS-700057 | -4.67 | 4.36 | -1.07 | 0.284 | 1.000 |
|  | LPS-RT001 | -9.67 | 4.36 | -2.22 | 0.027 | 0.399 |
|  | LPS-Un | 12.67 | 4.36 | 2.91 | 0.004 | 0.055 |
|  | 700057-RT001 | 5.00 | 4.36 | 1.15 | 0.251 | 1.000 |
|  | 700057-Un | 8.00 | 4.36 | 1.84 | 0.066 | 0.997 |
|  | RT001-Un | 3.00 | 4.36 | 0.69 | 0.491 | 1.000 |
|  | RT126-LPS | 1.00 | 4.36 | 0.23 | 0.819 | 1.000 |
|  | RT126-700057 | -5.67 | 4.36 | -1.30 | 0.194 | 1.000 |
|  | RT126-RT084 | -6.00 | 4.36 | -1.38 | 0.169 | 1.000 |
|  | RT126-RT001 | -10.67 | 4.36 | -2.45 | 0.014 | 0.216 |
|  | RT126-Un | 13.67 | 4.36 | 3.14 | 0.002 | 0.026 |
|  | LPS-RT084 | -5.00 | 4.36 | -1.15 | 0.251 | 1.000 |
|  | 700057-RT084 | 0.33 | 4.36 | 0.08 | 0.939 | 1.000 |
| **24 h** | RT084-LPS | 8.67 | 4.35 | 1.99 | 0.047 | 0.698 |
|  | RT084-700057 | -13.17 | 4.35 | -2.98 | 0.009 | 0.057 |
|  | RT084-RT001 | 3.00 | 4.35 | 0.69 | 0.491 | 1.000 |
|  | RT084-Un | 13.17 | 4.35 | 3.02 | 0.002 | 0.037 |
|  | RT084-RT126 | 5.00 | 4.35 | 1.15 | 0.251 | 1.000 |
|  | LPS-700057 | -4.50 | 4.35 | -1.03 | 0.301 | 1.000 |
|  | LPS-Un | 4.50 | 4.35 | 1.03 | 0.301 | 1.000 |
|  | RT001-Un | 10.17 | 4.35 | 2.33 | 0.020 | 0.293 |
|  | RT001-RT126 | 2.00 | 4.35 | 0.46 | 0.646 | 1.000 |
|  | RT126-LPS | 3.67 | 4.35 | 0.84 | 0.400 | 1.000 |
|  | RT126-700057 | -8.17 | 4.35 | -1.88 | 0.061 | 0.911 |
|  | RT126-Un | 8.17 | 4.35 | 1.88 | 0.061 | 0.911 |
|  | RT001-LPS | 5.67 | 4.35 | 1.30 | 0.193 | 1.000 |
|  | RT001-700057 | -10.17 | 4.35 | -2.33 | 0.020 | 0.293 |
|  | Un-700057 | 0.00 | 4.35 | 0.00 | 1.000 | 1.000 |

The multiple comparisons were not presented (the null hypothesis was retained) for time 8 h and therefore asymptotic significance is displayed.

**(D)**

| **Hour** | **Contrast** | **Test Statistic** | **Std. Error** | **Std. Test Statistic** | ***P*-value** | **Adj *P*-value^a^** |
| --- | --- | --- | --- | --- | --- | --- |
| **4 h** | RT001-RT126 | 0.167 | 4.334 | 0.038 | 0.969 | 1.000 |
|  | RT001-LPS | 2.833 | 4.334 | 0.654 | 0.513 | 1.000 |
|  | RT001-RT084 | -4.167 | 4.334 | -0.961 | 0.336 | 1.000 |
|  | RT001-700057 | -8.667 | 4.334 | -2.000 | 0.046 | 0.683 |
|  | RT001-Un | 12.167 | 4.334 | 2.807 | 0.005 | 0.075 |
|  | RT126-LPS | 2.667 | 4.334 | 0.615 | 0.538 | 1.000 |
|  | RT126-RT084 | -4.000 | 4.334 | -0.923 | 0.356 | 1.000 |
|  | RT126-700057 | -8.500 | 4.334 | -1.961 | 0.050 | 0.748 |
|  | RT126-Un | 12.000 | 4.334 | 2.769 | 0.006 | 0.084 |
|  | LPS-RT084 | -1.333 | 4.334 | -0.308 | 0.758 | 1.000 |
|  | LPS-700057 | -5.833 | 4.334 | -1.346 | 0.178 | 1.000 |
|  | LPS-Un | 9.333 | 4.334 | 2.153 | 0.031 | 0.469 |
|  | RT084-700057 | -4.500 | 4.334 | -1.038 | 0.299 | 1.000 |
|  | RT084-Un | 8.000 | 4.334 | 1.846 | 0.065 | 0.974 |
|  | 700057-Un | 3.500 | 4.334 | 0.808 | 0.419 | 1.000 |
| **8 h** | RT001-RT126 | 1.500 | 4.354 | 0.344 | 0.730 | 1.000 |
|  | RT001-RT084 | -2.333 | 4.354 | -0.536 | 0.592 | 1.000 |
|  | RT001-700057 | -10.500 | 4.354 | -2.411 | 0.016 | 0.238 |
|  | RT001-Un | 6.000 | 4.354 | 1.378 | 0.168 | 1.000 |
|  | RT126-RT084 | -0.833 | 4.354 | -0.191 | 0.848 | 1.000 |
|  | RT126-700057 | -9.000 | 4.354 | -2.067 | 0.039 | 0.581 |
|  | RT126-Un | 4.500 | 4.354 | 1.033 | 0.301 | 1.000 |
|  | LPS-RT084 | -4.667 | 4.354 | -1.072 | 0.284 | 1.000 |
|  | LPS-700057 | -12.833 | 4.354 | -2.747 | 0.011 | 0.058 |
|  | LPS-Un | 8.333 | 4.354 | 1.914 | 0.056 | 0.835 |
|  | RT084-700057 | -8.167 | 4.354 | -1.875 | 0.061 | 0.911 |
|  | RT084-Un | 3.667 | 4.354 | 0.842 | 0.400 | 1.000 |
|  | LPS-RT001 | -2.333 | 4.354 | -0.536 | 0.592 | 1.000 |
|  | LPS-RT126 | -3.833 | 4.354 | -0.880 | 0.379 | 1.000 |
|  | Un-700057 | -4.500 | 4.354 | -1.033 | 0.301 | 1.000 |
| **12 h** | RT001-LPS | 1.333 | 4.348 | 0.307 | 0.759 | 1.000 |
|  | RT001-700057 | -6.667 | 4.348 | -1.533 | 0.125 | 1.000 |
|  | RT001-Un | 9.333 | 4.348 | 2.147 | 0.032 | 0.477 |
|  | RT126-LPS | 3.333 | 4.348 | 0.767 | 0.443 | 1.000 |
|  | RT126-700057 | -8.667 | 4.348 | -1.993 | 0.046 | 0.693 |
|  | RT126-Un | 11.333 | 4.348 | 2.607 | 0.009 | 0.137 |
|  | LPS-700057 | -5.333 | 4.348 | -1.227 | 0.220 | 1.000 |
|  | LPS-Un | 8.000 | 4.348 | 1.840 | 0.066 | 0.986 |
|  | RT084-700057 | -10.000 | 4.348 | -2.300 | 0.021 | 0.322 |
|  | RT084-Un | 12.667 | 4.348 | 2.913 | 0.004 | 0.054 |
|  | 700057-Un | 2.667 | 4.348 | 0.613 | 0.540 | 1.000 |
|  | RT084-RT126 | 1.333 | 4.348 | 0.307 | 0.759 | 1.000 |
|  | RT084-RT001 | 3.333 | 4.348 | 0.767 | 0.443 | 1.000 |
|  | RT084-LPS | 4.667 | 4.348 | 1.073 | 0.283 | 1.000 |
|  | RT126-RT001 | -2.000 | 4.348 | -0.460 | 0.646 | 1.000 |
| **24 h** | RT001-RT084 | -4.167 | 4.354 | -0.957 | 0.339 | 1.000 |
|  | RT001-700057 | -1.833 | 4.354 | -0.421 | 0.674 | 1.000 |
|  | RT001-Un | 3.333 | 4.354 | 0.766 | 0.444 | 1.000 |
|  | RT126-RT084 | -9.500 | 4.354 | -2.182 | 0.029 | 0.437 |
|  | RT126-700057 | -7.167 | 4.354 | -1.646 | 0.100 | 1.000 |
|  | RT126-Un | 8.667 | 4.354 | 1.990 | 0.047 | 0.698 |
|  | LPS-RT084 | -11.167 | 4.354 | -2.564 | 0.010 | 0.155 |
|  | LPS-700057 | -8.833 | 4.354 | -2.029 | 0.042 | 0.637 |
|  | LPS-Un | 10.333 | 4.354 | 2.373 | 0.018 | 0.265 |
|  | 700057-Un | 1.500 | 4.354 | 0.344 | 0.730 | 1.000 |
|  | LPS-RT001 | -7.000 | 4.354 | -1.608 | 0.108 | 1.000 |
|  | LPS-RT126 | -1.667 | 4.354 | -0.383 | 0.702 | 1.000 |
|  | RT126-RT001 | -5.333 | 4.354 | -1.225 | 0.221 | 1.000 |
|  | 700057-RT084 | 2.333 | 4.354 | 0.536 | 0.592 | 1.000 |
|  | Un-RT084 | -0.833 | 4.354 | -0.191 | 0.848 | 1.000 |

**(E)**

| **Hour** | **Contrast** | **Test Statistic** | **Std. Error** | **Std. Test Statistic** | ***P*-value** | **Adj *P*-value^a^** |
| --- | --- | --- | --- | --- | --- | --- |
| **8 h** | RT126-RT084 | -3.50 | 4.35 | -0.80 | 0.421 | 1.000 |
|  | RT126-LPS | 5.33 | 4.35 | 1.23 | 0.220 | 1.000 |
|  | RT126-RT001 | -8.83 | 4.35 | -2.03 | 0.042 | 0.634 |
|  | RT126-700057 | -9.83 | 4.35 | -2.26 | 0.024 | 0.357 |
|  | RT126-Un | 14.50 | 4.35 | 3.33 | 0.001 | 0.013 |
|  | RT084-LPS | 1.83 | 4.35 | 0.42 | 0.673 | 1.000 |
|  | RT084-RT001 | 5.33 | 4.35 | 1.23 | 0.220 | 1.000 |
|  | RT084-700057 | -6.33 | 4.35 | -1.46 | 0.145 | 1.000 |
|  | RT084-Un | 11.00 | 4.35 | 2.53 | 0.011 | 0.172 |
|  | LPS-RT001 | -3.50 | 4.35 | -0.80 | 0.421 | 1.000 |
|  | LPS-700057 | -4.50 | 4.35 | -1.03 | 0.301 | 1.000 |
|  | LPS-Un | 9.17 | 4.35 | 2.11 | 0.035 | 0.526 |
|  | RT001-700057 | -1.00 | 4.35 | -0.23 | 0.818 | 1.000 |
|  | RT001-Un | 5.67 | 4.35 | 1.30 | 0.193 | 1.000 |
|  | 700057-Un | 4.67 | 4.35 | 1.07 | 0.283 | 1.000 |
| **12 h** | RT126-RT084 | -3.17 | 4.33 | -0.73 | 0.465 | 1.000 |
|  | RT126-RT001 | -6.50 | 4.33 | -1.50 | 0.133 | 1.000 |
|  | RT126-700057 | -3.33 | 4.33 | -0.77 | 0.442 | 1.000 |
|  | RT126-Un | 11.33 | 4.33 | 2.62 | 0.009 | 0.133 |
|  | RT084-RT001 | 3.33 | 4.33 | 0.77 | 0.442 | 1.000 |
|  | RT084-700057 | -0.17 | 4.33 | -0.04 | 0.969 | 1.000 |
|  | RT084-Un | 8.17 | 4.33 | 1.89 | 0.059 | 0.891 |
|  | LPS-RT001 | -7.83 | 4.33 | -1.81 | 0.071 | 1.000 |
|  | LPS-700057 | -4.67 | 4.33 | -1.08 | 0.281 | 1.000 |
|  | LPS-Un | 12.67 | 4.33 | 2.92 | 0.003 | 0.052 |
|  | RT001-Un | 4.83 | 4.33 | 1.12 | 0.265 | 1.000 |
|  | 700057-Un | 8.00 | 4.33 | 1.85 | 0.065 | 0.972 |
|  | LPS-RT126 | -1.33 | 4.33 | -0.31 | 0.758 | 1.000 |
|  | LPS-RT084 | -4.50 | 4.33 | -1.04 | 0.299 | 1.000 |
|  | 700057-RT001 | 3.17 | 4.33 | 0.73 | 0.465 | 1.000 |
| **24 h** | RT126-RT084 | -7.67 | 4.36 | -1.76 | 0.079 | 1.000 |
|  | RT126-RT001 | -6.67 | 4.36 | -1.53 | 0.126 | 1.000 |
|  | RT126-700057 | -3.33 | 4.36 | -0.76 | 0.444 | 1.000 |
|  | RT126-Un | 12.00 | 4.36 | 2.75 | 0.006 | 0.089 |
|  | RT084-Un | 4.33 | 4.36 | 0.99 | 0.320 | 1.000 |
|  | LPS-RT001 | -9.33 | 4.36 | -2.14 | 0.032 | 0.484 |
|  | LPS-700057 | -6.00 | 4.36 | -1.38 | 0.169 | 1.000 |
|  | LPS-Un | 14.67 | 4.36 | 3.36 | 0.001 | 0.011 |
|  | RT001-Un | 5.33 | 4.36 | 1.22 | 0.221 | 1.000 |
|  | 700057-Un | 8.67 | 4.36 | 1.99 | 0.047 | 0.702 |
|  | LPS-RT126 | -2.67 | 4.36 | -0.61 | 0.541 | 1.000 |
|  | LPS-RT084 | -10.33 | 4.36 | -2.37 | 0.018 | 0.266 |
|  | 700057-RT001 | 3.33 | 4.36 | 0.76 | 0.444 | 1.000 |
|  | 700057-RT084 | 4.33 | 4.36 | 0.99 | 0.320 | 1.000 |
|  | RT001-RT084 | -1.00 | 4.36 | -0.23 | 0.819 | 1.000 |

The multiple comparisons were not presented (the null hypothesis was retained) for time 4 h and therefore asymptotic significance is displayed.

**(F)**

| **Hour** | **Contrast** | **Test Statistic** | **Std. Error** | **Std. Test Statistic** | ***P*-value** | **Adj *P*-value^a^** |
| --- | --- | --- | --- | --- | --- | --- |
| **8 h** | LPS-RT084 | -4.33 | 4.35 | -1.00 | 0.319 | 1.000 |
|  | LPS-700057 | -7.17 | 4.35 | -1.65 | 0.099 | 1.000 |
|  | LPS-RT001 | -7.67 | 4.35 | -1.76 | 0.078 | 1.000 |
|  | LPS-RT126 | -10.67 | 4.35 | -2.45 | 0.014 | 0.212 |
|  | LPS-Un | 14.17 | 4.35 | 3.26 | 0.001 | 0.017 |
|  | RT084-700057 | -2.83 | 4.35 | -0.65 | 0.515 | 1.000 |
|  | RT084-RT001 | 3.33 | 4.35 | 0.77 | 0.443 | 1.000 |
|  | RT084-RT126 | 6.33 | 4.35 | 1.46 | 0.145 | 1.000 |
|  | RT084-Un | 9.83 | 4.35 | 2.26 | 0.024 | 0.356 |
|  | 700057-RT001 | 0.50 | 4.35 | 0.12 | 0.908 | 1.000 |
|  | 700057-RT126 | 3.50 | 4.35 | 0.81 | 0.421 | 1.000 |
|  | 700057-Un | 7.00 | 4.35 | 1.61 | 0.107 | 1.000 |
|  | RT001-RT126 | 3.00 | 4.35 | 0.69 | 0.490 | 1.000 |
|  | RT001-Un | 6.50 | 4.35 | 1.50 | 0.135 | 1.000 |
|  | RT126-Un | 3.50 | 4.35 | 0.81 | 0.421 | 1.000 |
| **12 h** | LPS-700057 | -5.83 | 4.35 | -1.34 | 0.180 | 1.000 |
|  | LPS-RT001 | -9.33 | 4.35 | -2.14 | 0.032 | 0.481 |
|  | LPS-RT126 | -2.33 | 4.35 | -0.54 | 0.592 | 1.000 |
|  | LPS-Un | 12.33 | 4.35 | 2.23 | 0.005 | 0.069 |
|  | RT084-700057 | -6.67 | 4.35 | -1.53 | 0.126 | 1.000 |
|  | RT084-RT001 | 10.17 | 4.35 | 2.33 | 0.020 | 0.293 |
|  | RT084-RT126 | 3.17 | 4.35 | 0.73 | 0.467 | 1.000 |
|  | RT084-Un | 13.17 | 4.35 | 3.02 | 0.002 | 0.037 |
|  | 700057-RT001 | 3.50 | 4.35 | 0.80 | 0.422 | 1.000 |
|  | 700057-Un | 6.50 | 4.35 | 1.49 | 0.136 | 1.000 |
|  | RT001-Un | 3.00 | 4.35 | 0.69 | 0.491 | 1.000 |
|  | RT126-Un | 10.00 | 4.35 | 2.30 | 0.022 | 0.325 |
|  | RT084-LPS | 0.83 | 4.35 | 0.19 | 0.848 | 1.000 |
|  | RT126-700057 | -3.50 | 4.35 | -0.80 | 0.422 | 1.000 |
|  | RT126-RT001 | -7.00 | 4.35 | -1.61 | 0.108 | 1.000 |
| **24 h** | LPS-RT084 | -5.00 | 4.34 | -1.15 | 0.249 | 1.000 |
|  | LPS-700057 | -9.83 | 4.34 | -2.27 | 0.023 | 0.351 |
|  | LPS-RT001 | -5.00 | 4.34 | -1.15 | 0.249 | 1.000 |
|  | LPS-RT126 | -8.50 | 4.34 | -1.96 | 0.050 | 0.751 |
|  | LPS-Un | 14.67 | 4.34 | 3.38 | 0.001 | 0.011 |
|  | RT084-700057 | -4.83 | 4.34 | -1.11 | 0.265 | 1.000 |
|  | RT084-RT126 | 3.50 | 4.34 | 0.81 | 0.420 | 1.000 |
|  | RT084-Un | 9.67 | 4.34 | 2.23 | 0.026 | 0.388 |
|  | 700057-Un | 4.83 | 4.34 | 1.11 | 0.265 | 1.000 |
|  | RT001-RT126 | 3.50 | 4.34 | 0.81 | 0.420 | 1.000 |
|  | RT001-Un | 9.67 | 4.34 | 2.23 | 0.026 | 0.388 |
|  | RT126-Un | 6.17 | 4.34 | 1.42 | 0.155 | 1.000 |
|  | RT126-700057 | -1.33 | 4.34 | -0.31 | 0.759 | 1.000 |
|  | RT001-RT084 | 0.00 | 4.34 | 0.00 | 1.000 | 1.000 |
|  | RT001-700057 | -4.83 | 4.34 | -1.11 | 0.265 | 1.000 |

The multiple comparisons were not presented (the null hypothesis was retained) for time 4 h and therefore asymptotic significance is displayed.

**(G)**

| **Hour** | **Contrast** | **Test Statistic** | **Std. Error** | **Std. Test Statistic** | ***P*-value** | **Adj *P*-value^a^** |
| --- | --- | --- | --- | --- | --- | --- |
| **4 h** | LPS-RT001 | -2.33 | 4.36 | -0.54 | 0.592 | 1.000 |
|  | LPS-Un | 7.33 | 4.36 | 1.68 | 0.092 | 1.000 |
|  | LPS-RT126 | -7.67 | 4.36 | -1.76 | 0.078 | 1.000 |
|  | LPS-RT084 | -11.00 | 4.36 | -2.52 | 0.012 | 0.174 |
|  | LPS-700057 | -14.67 | 4.36 | -2.07 | 0.011 | 0.051 |
|  | RT001-Un | 5.00 | 4.36 | 1.15 | 0.251 | 1.000 |
|  | RT001-RT126 | 5.33 | 4.36 | 1.22 | 0.221 | 1.000 |
|  | RT001-RT084 | -8.67 | 4.36 | -1.99 | 0.047 | 0.700 |
|  | RT001-700057 | -12.33 | 4.36 | -2.83 | 0.005 | 0.070 |
|  | Un-RT126 | -0.33 | 4.36 | -0.08 | 0.939 | 1.000 |
|  | Un-RT084 | -3.67 | 4.36 | -0.84 | 0.400 | 1.000 |
|  | Un-700057 | -7.33 | 4.36 | -1.68 | 0.092 | 1.000 |
|  | RT126-RT084 | -3.33 | 4.36 | -0.77 | 0.444 | 1.000 |
|  | RT126-700057 | -7.00 | 4.36 | -1.61 | 0.108 | 1.000 |
|  | RT084-700057 | -3.67 | 4.36 | -0.84 | 0.400 | 1.000 |
| **8 h** | LPS-RT001 | -3.67 | 4.35 | -0.84 | 0.400 | 1.000 |
|  | LPS-Un | 14.67 | 4.35 | 3.37 | 0.001 | 0.061 |
|  | LPS-RT126 | -6.17 | 4.35 | -1.42 | 0.157 | 1.000 |
|  | LPS-RT084 | -8.33 | 4.35 | -1.91 | 0.056 | 0.835 |
|  | LPS-700057 | -12.17 | 4.35 | -2.79 | 0.005 | 0.078 |
|  | RT001-Un | 11.00 | 4.35 | 2.53 | 0.012 | 0.173 |
|  | RT001-RT126 | 2.50 | 4.35 | 0.57 | 0.566 | 1.000 |
|  | RT001-RT084 | -4.67 | 4.35 | -1.07 | 0.284 | 1.000 |
|  | RT001-700057 | -8.50 | 4.35 | -1.95 | 0.051 | 0.764 |
|  | RT126-RT084 | -2.17 | 4.35 | -0.50 | 0.619 | 1.000 |
|  | RT126-700057 | -6.00 | 4.35 | -1.38 | 0.168 | 1.000 |
|  | RT084-700057 | -3.83 | 4.35 | -0.88 | 0.379 | 1.000 |
|  | RT126-Un | 8.50 | 4.35 | 1.95 | 0.051 | 0.764 |
|  | RT084-Un | 6.33 | 4.35 | 1.45 | 0.146 | 1.000 |
|  | 700057-Un | 2.50 | 4.35 | 0.57 | 0.566 | 1.000 |
| **24 h** | LPS-RT001 | -4.50 | 4.36 | -1.03 | 0.302 | 1.000 |
|  | LPS-Un | 12.00 | 4.36 | 2.75 | 0.006 | 0.088 |
|  | LPS-RT126 | -5.67 | 4.36 | -1.30 | 0.193 | 1.000 |
|  | LPS-700057 | -11.00 | 4.36 | -2.52 | 0.012 | 0.174 |
|  | RT001-Un | 7.50 | 4.36 | 1.72 | 0.085 | 1.000 |
|  | RT001-RT126 | 1.17 | 4.36 | 0.27 | 0.789 | 1.000 |
|  | RT001-700057 | -6.50 | 4.36 | -1.49 | 0.136 | 1.000 |
|  | RT126-700057 | -5.33 | 4.36 | -1.22 | 0.221 | 1.000 |
|  | RT084-700057 | -11.17 | 4.36 | -2.56 | 0.010 | 0.156 |
|  | RT126-Un | 6.33 | 4.36 | 1.45 | 0.146 | 1.000 |
|  | RT084-Un | 12.17 | 4.36 | 2.79 | 0.005 | 0.078 |
|  | 700057-Un | 1.00 | 4.36 | 0.23 | 0.818 | 1.000 |
|  | RT084-LPS | 0.17 | 4.36 | 0.04 | 0.969 | 1.000 |
|  | RT084-RT001 | 4.67 | 4.36 | 1.07 | 0.284 | 1.000 |
|  | RT084-RT126 | 5.83 | 4.36 | 1.34 | 0.181 | 1.000 |

The multiple comparisons were not presented (the null hypothesis was retained) for time 12 h and therefore asymptotic significance is displayed.

**(H)**

| **Hour** | **Contrast** | **Test Statistic** | **Std. Error** | **Std. Test Statistic** | ***P*-value** | **Adj *P*-value^a^** |
| --- | --- | --- | --- | --- | --- | --- |
| **8 h** | LPS-RT126 | -5.67 | 4.35 | -1.30 | 0.193 | 1.000 |
|  | LPS-700057 | -7.00 | 4.35 | -1.61 | 0.108 | 1.000 |
|  | LPS-RT001 | -7.17 | 4.35 | -1.65 | 0.100 | 1.000 |
|  | LPS-RT084 | -10.50 | 4.35 | -2.41 | 0.016 | 0.238 |
|  | LPS-Un | 14.67 | 4.35 | 1.37 | 0.001 | 0.061 |
|  | RT126-700057 | -1.33 | 4.35 | -0.31 | 0.759 | 1.000 |
|  | RT126-RT001 | -1.50 | 4.35 | -0.34 | 0.730 | 1.000 |
|  | RT126-RT084 | -4.83 | 4.35 | -1.11 | 0.267 | 1.000 |
|  | RT126-Un | 9.00 | 4.35 | 2.07 | 0.039 | 0.581 |
|  | 700057-RT001 | 0.17 | 4.35 | 0.04 | 0.969 | 1.000 |
|  | 700057-RT084 | 3.50 | 4.35 | 0.80 | 0.422 | 1.000 |
|  | 700057-Un | 7.67 | 4.35 | 1.76 | 0.078 | 1.000 |
|  | RT001-RT084 | -3.33 | 4.35 | -0.77 | 0.444 | 1.000 |
|  | RT001-Un | 7.50 | 4.35 | 1.72 | 0.085 | 1.000 |
|  | RT084-Un | 4.17 | 4.35 | 0.96 | 0.339 | 1.000 |
| **12 h** | LPS-RT126 | -2.83 | 4.36 | -0.65 | 0.515 | 1.000 |
|  | LPS-RT001 | -8.33 | 4.36 | -1.91 | 0.056 | 0.837 |
|  | LPS-RT084 | -1.50 | 4.36 | -0.34 | 0.731 | 1.000 |
|  | LPS-Un | 11.50 | 4.36 | 2.64 | 0.008 | 0.124 |
|  | RT126-RT001 | -5.50 | 4.36 | -1.26 | 0.207 | 1.000 |
|  | RT126-Un | 8.67 | 4.36 | 1.99 | 0.047 | 0.700 |
|  | 700057-RT001 | 8.50 | 4.36 | 1.95 | 0.051 | 0.766 |
|  | 700057-RT084 | 1.67 | 4.36 | 0.38 | 0.702 | 1.000 |
|  | 700057-Un | 11.67 | 4.36 | 2.68 | 0.007 | 0.111 |
|  | RT001-Un | 3.17 | 4.36 | 0.73 | 0.467 | 1.000 |
|  | RT084-Un | 10.00 | 4.36 | 2.30 | 0.022 | 0.326 |
|  | 700057-LPS | 0.17 | 4.36 | 0.04 | 0.969 | 1.000 |
|  | 700057-RT126 | 3.00 | 4.36 | 0.69 | 0.491 | 1.000 |
|  | RT084-RT126 | 1.33 | 4.36 | 0.31 | 0.760 | 1.000 |
|  | RT084-RT001 | 6.83 | 4.36 | 1.57 | 0.117 | 1.000 |
| **24 h** | LPS-RT126 | -3.00 | 4.35 | -0.69 | 0.491 | 1.000 |
|  | LPS-700057 | -10.33 | 4.35 | -2.37 | 0.018 | 0.264 |
|  | LPS-RT001 | -6.50 | 4.35 | -1.49 | 0.135 | 1.000 |
|  | LPS-RT084 | -5.17 | 4.35 | -1.19 | 0.235 | 1.000 |
|  | LPS-Un | 14.00 | 4.35 | 3.22 | 0.131 | 0.169 |
|  | RT126-700057 | -7.33 | 4.35 | -1.68 | 0.092 | 1.000 |
|  | RT126-RT001 | -3.50 | 4.35 | -0.80 | 0.421 | 1.000 |
|  | RT126-RT084 | -2.17 | 4.35 | -0.50 | 0.619 | 1.000 |
|  | RT126-Un | 11.00 | 4.35 | 2.53 | 0.011 | 0.172 |
|  | 700057-Un | 3.67 | 4.35 | 0.84 | 0.400 | 1.000 |
|  | RT001-Un | 7.50 | 4.35 | 1.72 | 0.085 | 1.000 |
|  | RT084-Un | 8.83 | 4.35 | 2.03 | 0.042 | 0.636 |
|  | RT084-RT001 | 1.33 | 4.35 | 0.31 | 0.759 | 1.000 |
|  | RT084-700057 | -5.17 | 4.35 | -1.19 | 0.235 | 1.000 |
|  | RT001-700057 | -3.83 | 4.35 | -0.88 | 0.378 | 1.000 |

The multiple comparisons were not presented (the null hypothesis was retained) for time 4 h and therefore asymptotic significance is displayed.

**(I)**

| **Hour** | **Contrast** | **Test Statistic** | **Std. Error** | **Std. Test Statistic** | ***P*-value** | **Adj *P*-value^a^** |
| --- | --- | --- | --- | --- | --- | --- |
| **4 h** | Un-RT126 | -3.67 | 4.36 | -0.84 | 0.400 | 1.000 |
|  | Un-700057 | -5.33 | 4.36 | -1.22 | 0.221 | 1.000 |
|  | Un-RT084 | -10.67 | 4.36 | -2.45 | 0.014 | 0.215 |
|  | Un-RT001 | -11.67 | 4.36 | -2.68 | 0.007 | 0.111 |
|  | Un-LPS | -13.67 | 4.36 | -3.14 | 0.002 | 0.026 |
|  | RT126-700057 | -1.67 | 4.36 | -0.38 | 0.702 | 1.000 |
|  | RT126-RT084 | -7.00 | 4.36 | -1.61 | 0.108 | 1.000 |
|  | RT126-RT001 | -8.00 | 4.36 | -1.84 | 0.066 | 0.995 |
|  | RT126-LPS | 10.00 | 4.36 | 2.30 | 0.022 | 0.326 |
|  | 700057-RT084 | 5.33 | 4.36 | 1.22 | 0.221 | 1.000 |
|  | 700057-RT001 | 6.33 | 4.36 | 1.45 | 0.146 | 1.000 |
|  | 700057-LPS | 8.33 | 4.36 | 1.91 | 0.056 | 0.837 |
|  | RT084-RT001 | 1.00 | 4.36 | 0.23 | 0.818 | 1.000 |
|  | RT084-LPS | 3.00 | 4.36 | 0.69 | 0.491 | 1.000 |
|  | RT001-LPS | 2.00 | 4.36 | 0.46 | 0.646 | 1.000 |
| **8 h** | Un-700057 | -9.67 | 4.36 | -2.22 | 0.027 | 0.399 |
|  | Un-RT084 | -6.67 | 4.36 | -1.53 | 0.126 | 1.000 |
|  | Un-RT001 | -6.33 | 4.36 | -1.45 | 0.146 | 1.000 |
|  | Un-LPS | -12.67 | 4.36 | -2.91 | 0.004 | 0.055 |
|  | RT126-700057 | -10.00 | 4.36 | -2.29 | 0.022 | 0.327 |
|  | RT126-RT084 | -7.00 | 4.36 | -1.61 | 0.108 | 1.000 |
|  | RT126-RT001 | -6.67 | 4.36 | -1.53 | 0.126 | 1.000 |
|  | 700057-LPS | 3.00 | 4.36 | 0.69 | 0.491 | 1.000 |
|  | RT084-LPS | 6.00 | 4.36 | 1.38 | 0.169 | 1.000 |
|  | RT001-LPS | 6.33 | 4.36 | 1.45 | 0.146 | 1.000 |
|  | RT126-Un | 0.33 | 4.36 | 0.08 | 0.939 | 1.000 |
|  | RT001-RT084 | -0.33 | 4.36 | -0.08 | 0.939 | 1.000 |
|  | RT001-700057 | -3.33 | 4.36 | -0.76 | 0.444 | 1.000 |
|  | RT084-700057 | -3.00 | 4.36 | -0.69 | 0.491 | 1.000 |
| **12 h** | Un-RT126 | -12.00 | 4.36 | -2.75 | 0.006 | 0.088 |
|  | Un-700057 | -1.00 | 4.36 | -0.23 | 0.818 | 1.000 |
|  | Un-RT084 | -5.00 | 4.36 | -1.15 | 0.251 | 1.000 |
|  | Un-RT001 | -8.00 | 4.36 | -1.84 | 0.066 | 0.995 |
|  | Un-LPS | -13.00 | 4.36 | -2.98 | 0.003 | 0.043 |
|  | RT126-LPS | 1.00 | 4.36 | 0.23 | 0.818 | 1.000 |
|  | 700057-RT084 | 4.00 | 4.36 | 0.92 | 0.359 | 1.000 |
|  | 700057-RT001 | 7.00 | 4.36 | 1.61 | 0.108 | 1.000 |
|  | 700057-LPS | 12.00 | 4.36 | 2.75 | 0.006 | 0.088 |
|  | RT084-RT001 | 3.00 | 4.36 | 0.69 | 0.491 | 1.000 |
|  | RT084-LPS | 8.00 | 4.36 | 1.84 | 0.066 | 0.995 |
|  | RT001-LPS | 5.00 | 4.36 | 1.15 | 0.251 | 1.000 |
|  | 700057-RT126 | 11.00 | 4.36 | 2.52 | 0.012 | 0.174 |
|  | RT084-RT126 | 7.00 | 4.36 | 1.61 | 0.108 | 1.000 |
|  | RT001-RT126 | 4.00 | 4.36 | 0.92 | 0.359 | 1.000 |
| **24 h** | Un-RT126 | -12.67 | 4.35 | -2.91 | 0.004 | 0.054 |
|  | Un-700057 | -3.50 | 4.35 | -0.80 | 0.422 | 1.000 |
|  | Un-RT084 | -5.50 | 4.35 | -1.26 | 0.207 | 1.000 |
|  | Un-RT001 | -9.00 | 4.35 | -2.07 | 0.039 | 0.581 |
|  | Un-LPS | -14.33 | 4.35 | -3.29 | 0.001 | 0.015 |
|  | RT126-LPS | 1.67 | 4.35 | 0.38 | 0.702 | 1.000 |
|  | 700057-RT084 | 2.00 | 4.35 | 0.46 | 0.646 | 1.000 |
|  | 700057-RT001 | 5.50 | 4.35 | 1.26 | 0.207 | 1.000 |
|  | 700057-LPS | 10.83 | 4.35 | 2.49 | 0.013 | 0.193 |
|  | RT084-RT001 | 3.50 | 4.35 | 0.80 | 0.422 | 1.000 |
|  | RT084-LPS | 8.83 | 4.35 | 2.03 | 0.042 | 0.637 |
|  | RT001-LPS | 5.33 | 4.35 | 1.22 | 0.221 | 1.000 |
|  | 700057-RT126 | 9.17 | 4.35 | 2.11 | 0.035 | 0.529 |
|  | RT084-RT126 | 7.17 | 4.35 | 1.65 | 0.100 | 1.000 |
|  | RT001-RT126 | 3.67 | 4.35 | 0.84 | 0.400 | 1.000 |

**(J)**

| **Hour** | **Contrast** | **Test Statistic** | **Std. Error** | **Std. Test Statistic** | ***P*-value** | **Adj *P*-value^a^** |
| --- | --- | --- | --- | --- | --- | --- |
| **4 h** | Un-RT126 | -3.67 | 4.36 | -0.84 | 0.400 | 1.000 |
|  | Un-700057 | -5.33 | 4.36 | -1.22 | 0.221 | 1.000 |
|  | Un-RT084 | -10.00 | 4.36 | -2.30 | 0.022 | 0.326 |
|  | Un-LPS | -12.67 | 4.36 | -2.91 | 0.004 | 0.055 |
|  | Un-RT001 | -13.33 | 4.36 | -3.06 | 0.002 | 0.033 |
|  | RT126-700057 | -1.67 | 4.36 | -0.38 | 0.702 | 1.000 |
|  | RT126-RT084 | -6.33 | 4.36 | -1.45 | 0.146 | 1.000 |
|  | RT126-LPS | 9.00 | 4.36 | 2.07 | 0.039 | 0.583 |
|  | RT126-RT001 | -9.67 | 4.36 | -2.22 | 0.026 | 0.397 |
|  | 700057-RT084 | 4.67 | 4.36 | 1.07 | 0.284 | 1.000 |
|  | 700057-LPS | 7.33 | 4.36 | 1.68 | 0.092 | 1.000 |
|  | 700057-RT001 | 8.00 | 4.36 | 1.84 | 0.066 | 0.995 |
|  | RT084-LPS | 2.67 | 4.36 | 0.61 | 0.540 | 1.000 |
|  | RT084-RT001 | 3.33 | 4.36 | 0.77 | 0.444 | 1.000 |
|  | LPS-RT001 | -0.67 | 4.36 | -0.15 | 0.878 | 1.000 |
| **8 h** | Un-700057 | -8.83 | 4.36 | -2.03 | 0.043 | 0.639 |
|  | Un-RT084 | -5.67 | 4.36 | -1.30 | 0.193 | 1.000 |
|  | Un-LPS | -12.00 | 4.36 | -2.75 | 0.006 | 0.088 |
|  | Un-RT001 | -8.83 | 4.36 | -2.03 | 0.043 | 0.639 |
|  | RT126-700057 | -9.17 | 4.36 | -2.10 | 0.035 | 0.531 |
|  | RT126-RT084 | -6.00 | 4.36 | -1.38 | 0.168 | 1.000 |
|  | RT126-LPS | 12.33 | 4.36 | 2.83 | 0.005 | 0.070 |
|  | RT126-RT001 | -9.17 | 4.36 | -2.10 | 0.035 | 0.531 |
|  | 700057-LPS | 3.17 | 4.36 | 0.73 | 0.467 | 1.000 |
|  | RT084-LPS | 6.33 | 4.36 | 1.45 | 0.146 | 1.000 |
|  | RT084-RT001 | 3.17 | 4.36 | 0.73 | 0.467 | 1.000 |
|  | RT126-Un | 0.33 | 4.36 | 0.08 | 0.939 | 1.000 |
|  | RT084-700057 | -3.17 | 4.36 | -0.73 | 0.467 | 1.000 |
|  | RT001-LPS | 3.17 | 4.36 | 0.73 | 0.467 | 1.000 |
|  | RT001-700057 | 0.00 | 4.36 | 0.00 | 1.000 | 1.000 |
| **12 h** | Un-RT126 | -10.67 | 4.36 | -2.45 | 0.014 | 0.215 |
|  | Un-700057 | -1.00 | 4.36 | -0.23 | 0.818 | 1.000 |
|  | Un-RT084 | -5.00 | 4.36 | -1.15 | 0.251 | 1.000 |
|  | Un-LPS | -12.00 | 4.36 | -2.75 | 0.006 | 0.088 |
|  | Un-RT001 | -10.33 | 4.36 | -2.37 | 0.018 | 0.265 |
|  | RT126-LPS | 1.33 | 4.36 | 0.31 | 0.760 | 1.000 |
|  | 700057-RT084 | 4.00 | 4.36 | 0.92 | 0.359 | 1.000 |
|  | 700057-LPS | 11.00 | 4.36 | 2.52 | 0.012 | 0.174 |
|  | 700057-RT001 | 9.33 | 4.36 | 2.14 | 0.032 | 0.483 |
|  | RT084-LPS | 7.00 | 4.36 | 1.61 | 0.108 | 1.000 |
|  | RT084-RT001 | 5.33 | 4.36 | 1.22 | 0.221 | 1.000 |
|  | RT001-LPS | 1.67 | 4.36 | 0.38 | 0.702 | 1.000 |
|  | 700057-RT126 | 9.67 | 4.36 | 2.22 | 0.026 | 0.397 |
|  | RT084-RT126 | 5.67 | 4.36 | 1.30 | 0.193 | 1.000 |
|  | RT001-RT126 | 0.33 | 4.36 | 0.08 | 0.939 | 1.000 |
| **24 h** | Un-RT126 | -12.00 | 4.35 | -2.76 | 0.006 | 0.088 |
|  | Un-700057 | -3.50 | 4.35 | -0.80 | 0.422 | 1.000 |
|  | Un-RT084 | -5.50 | 4.35 | -1.26 | 0.207 | 1.000 |
|  | Un-LPS | -15.00 | 4.35 | -3.44 | 0.001 | 0.009 |
|  | Un-RT001 | -9.00 | 4.35 | -2.07 | 0.039 | 0.581 |
|  | RT126-LPS | 3.00 | 4.35 | 0.69 | 0.491 | 1.000 |
|  | 700057-RT084 | 2.00 | 4.35 | 0.46 | 0.646 | 1.000 |
|  | 700057-LPS | 11.50 | 4.35 | 2.64 | 0.008 | 0.124 |
|  | 700057-RT001 | 5.50 | 4.35 | 1.26 | 0.207 | 1.000 |
|  | RT084-LPS | 9.50 | 4.35 | 2.18 | 0.029 | 0.437 |
|  | RT084-RT001 | 3.50 | 4.35 | 0.80 | 0.422 | 1.000 |
|  | RT001-LPS | 6.00 | 4.35 | 1.38 | 0.168 | 1.000 |
|  | 700057-RT126 | 8.50 | 4.35 | 1.95 | 0.051 | 0.764 |
|  | RT084-RT126 | 6.50 | 4.35 | 1.49 | 0.136 | 1.000 |
|  | RT001-RT126 | 3.00 | 4.35 | 0.69 | 0.491 | 1.000 |

**(K)**

| **Hour** | **Contrast** | **Test Statistic** | **Std. Error** | **Std. Test Statistic** | ***P*-value** | **Adj *P*-value^a^** |
| --- | --- | --- | --- | --- | --- | --- |
| **4 h** | Un-700057 | -5.333 | 4.357 | -1.224 | 0.221 | 1.000 |
|  | Un-RT126 | -5.833 | 4.357 | -1.339 | 0.181 | 1.000 |
|  | Un-RT001 | -8.167 | 4.357 | -1.875 | 0.061 | 0.913 |
|  | Un-RT084 | -9.833 | 4.357 | -2.257 | 0.024 | 0.360 |
|  | Un-LPS | -14.833 | 4.357 | -3.405 | 0.001 | 0.010 |
|  | 700057-RT126 | 0.500 | 4.357 | 0.115 | 0.909 | 1.000 |
|  | 700057-RT001 | 2.833 | 4.357 | 0.650 | 0.515 | 1.000 |
|  | 700057-RT084 | 4.500 | 4.357 | 1.033 | 0.302 | 1.000 |
|  | 700057-LPS | 9.500 | 4.357 | 2.181 | 0.029 | 0.438 |
|  | RT126-RT001 | -2.333 | 4.357 | -0.536 | 0.592 | 1.000 |
|  | RT126-RT084 | -4.000 | 4.357 | -0.918 | 0.359 | 1.000 |
|  | RT126-LPS | 9.000 | 4.357 | 2.066 | 0.039 | 0.583 |
|  | RT001-RT084 | -1.667 | 4.357 | -0.383 | 0.702 | 1.000 |
|  | RT001-LPS | 6.667 | 4.357 | 1.530 | 0.126 | 1.000 |
|  | RT084-LPS | 5.000 | 4.357 | 1.148 | 0.251 | 1.000 |
| **8 h** | Un-700057 | -3.667 | 4.359 | -0.841 | 0.400 | 1.000 |
|  | Un-RT126 | -10.000 | 4.359 | -2.294 | 0.022 | 0.327 |
|  | Un-RT001 | -6.667 | 4.359 | -1.529 | 0.126 | 1.000 |
|  | Un-RT084 | -14.667 | 4.359 | -3.365 | 0.001 | 0.011 |
|  | Un-LPS | -10.000 | 4.359 | -2.294 | 0.022 | 0.327 |
|  | 700057-RT126 | 6.333 | 4.359 | 1.453 | 0.146 | 1.000 |
|  | 700057-RT001 | 3.000 | 4.359 | 0.688 | 0.491 | 1.000 |
|  | 700057-RT084 | 11.000 | 4.359 | 2.524 | 0.012 | 0.174 |
|  | 700057-LPS | 6.333 | 4.359 | 1.453 | 0.146 | 1.000 |
|  | RT126-RT084 | -4.667 | 4.359 | -1.071 | 0.284 | 1.000 |
|  | RT001-RT084 | -8.000 | 4.359 | -1.835 | 0.066 | 0.997 |
|  | RT001-LPS | 3.333 | 4.359 | 0.765 | 0.444 | 1.000 |
|  | RT001-RT126 | 3.333 | 4.359 | 0.765 | 0.444 | 1.000 |
|  | LPS-RT126 | 0.000 | 4.359 | 0.000 | 1.000 | 1.000 |
|  | LPS-RT084 | -4.667 | 4.359 | -1.071 | 0.284 | 1.000 |
| **12 h** | Un-700057 | -3.667 | 4.359 | -0.841 | 0.400 | 1.000 |
|  | Un-RT126 | -14.667 | 4.359 | -3.365 | 0.001 | 0.011 |
|  | Un-RT001 | -12.333 | 4.359 | -2.829 | 0.005 | 0.070 |
|  | Un-RT084 | -7.333 | 4.359 | -1.682 | 0.092 | 1.000 |
|  | Un-LPS | -7.000 | 4.359 | -1.606 | 0.108 | 1.000 |
|  | 700057-RT126 | 11.000 | 4.359 | 2.524 | 0.012 | 0.174 |
|  | 700057-RT001 | 8.667 | 4.359 | 1.988 | 0.047 | 0.702 |
|  | 700057-RT084 | 3.667 | 4.359 | 0.841 | 0.400 | 1.000 |
|  | 700057-LPS | 3.333 | 4.359 | 0.765 | 0.444 | 1.000 |
|  | RT001-RT126 | 2.333 | 4.359 | 0.535 | 0.592 | 1.000 |
|  | LPS-RT126 | -7.667 | 4.359 | -1.759 | 0.079 | 1.000 |
|  | LPS-RT084 | -0.333 | 4.359 | -0.076 | 0.939 | 1.000 |
|  | LPS-RT001 | -5.333 | 4.359 | -1.224 | 0.221 | 1.000 |
|  | RT084-RT001 | 5.000 | 4.359 | 1.147 | 0.251 | 1.000 |
|  | RT084-RT126 | 7.333 | 4.359 | 1.682 | 0.092 | 1.000 |
| **24 h** | Un-700057 | -4.000 | 4.359 | -0.918 | 0.359 | 1.000 |
|  | Un-RT126 | -15.000 | 4.359 | -3.441 | 0.001 | 0.009 |
|  | Un-RT001 | -11.333 | 4.359 | -2.600 | 0.009 | 0.140 |
|  | Un-RT084 | -6.667 | 4.359 | -1.529 | 0.126 | 1.000 |
|  | Un-LPS | -8.000 | 4.359 | -1.835 | 0.066 | 0.997 |
|  | 700057-RT126 | 11.000 | 4.359 | 2.524 | 0.012 | 0.174 |
|  | 700057-RT001 | 7.333 | 4.359 | 1.682 | 0.092 | 1.000 |
|  | 700057-RT084 | 2.667 | 4.359 | 0.612 | 0.541 | 1.000 |
|  | 700057-LPS | 4.000 | 4.359 | 0.918 | 0.359 | 1.000 |
|  | RT084-LPS | 1.333 | 4.359 | 0.306 | 0.760 | 1.000 |
|  | RT001-RT126 | 3.667 | 4.359 | 0.841 | 0.400 | 1.000 |
|  | LPS-RT126 | -7.000 | 4.359 | -1.606 | 0.108 | 1.000 |
|  | LPS-RT001 | -3.333 | 4.359 | -0.765 | 0.444 | 1.000 |
|  | RT084-RT001 | 4.667 | 4.359 | 1.071 | 0.284 | 1.000 |
|  | RT084-RT126 | 8.333 | 4.359 | 1.912 | 0.056 | 0.839 |

**(L)**

| **Hour** | **Contrast** | **Test Statistic** | **Std. Error** | **Std. Test Statistic** | ***P*-value** | **Adj *P*-value^a^** |
| --- | --- | --- | --- | --- | --- | --- |
| **4 h** | RT084-RT001 | 2.67 | 4.35 | 0.61 | 0.539 | 1.000 |
|  | RT084-RT126 | 3.50 | 4.35 | 0.81 | 0.421 | 1.000 |
|  | RT084-Un | 8.67 | 4.35 | 1.99 | 0.046 | 0.692 |
|  | RT084-700057 | -10.83 | 4.35 | -2.49 | 0.013 | 0.190 |
|  | RT084-LPS | 12.33 | 4.35 | 2.84 | 0.005 | 0.068 |
|  | RT001-RT126 | 0.83 | 4.35 | 0.19 | 0.848 | 1.000 |
|  | RT001-Un | 6.00 | 4.35 | 1.38 | 0.167 | 1.000 |
|  | RT001-700057 | -8.17 | 4.35 | -1.88 | 0.060 | 0.903 |
|  | RT001-LPS | 9.67 | 4.35 | 2.22 | 0.026 | 0.392 |
|  | RT126-Un | 5.17 | 4.35 | 1.19 | 0.234 | 1.000 |
|  | RT126-700057 | -7.33 | 4.35 | -1.69 | 0.091 | 1.000 |
|  | RT126-LPS | 8.83 | 4.35 | 2.03 | 0.042 | 0.631 |
|  | Un-700057 | -2.17 | 4.35 | -0.50 | 0.618 | 1.000 |
|  | Un-LPS | -3.67 | 4.35 | -0.84 | 0.399 | 1.000 |
|  | 700057-LPS | 1.50 | 4.35 | 0.35 | 0.730 | 1.000 |
| **8 h** | RT084-RT001 | 6.33 | 4.35 | 1.46 | 0.145 | 1.000 |
|  | RT084-RT126 | 9.67 | 4.35 | 2.22 | 0.026 | 0.394 |
|  | RT084-LPS | 4.67 | 4.35 | 1.07 | 0.283 | 1.000 |
|  | RT001-RT126 | 3.33 | 4.35 | 0.77 | 0.443 | 1.000 |
|  | Un-700057 | -4.33 | 4.35 | -1.00 | 0.319 | 1.000 |
|  | Un-LPS | -9.67 | 4.35 | -2.22 | 0.026 | 0.394 |
|  | 700057-LPS | 5.33 | 4.35 | 1.23 | 0.220 | 1.000 |
|  | Un-RT084 | -5.00 | 4.35 | -1.15 | 0.250 | 1.000 |
|  | Un-RT001 | -11.33 | 4.35 | -2.61 | 0.009 | 0.138 |
|  | Un-RT126 | -14.67 | 4.35 | -3.37 | 0.001 | 0.011 |
|  | 700057-RT084 | 0.67 | 4.35 | 0.15 | 0.878 | 1.000 |
|  | 700057-RT001 | 7.00 | 4.35 | 1.61 | 0.108 | 1.000 |
|  | 700057-RT126 | 10.33 | 4.35 | 2.38 | 0.018 | 0.263 |
|  | LPS-RT001 | -1.67 | 4.35 | -0.38 | 0.702 | 1.000 |
|  | LPS-RT126 | -5.00 | 4.35 | -1.15 | 0.250 | 1.000 |
| **12 h** | RT084-RT126 | 1.83 | 4.35 | 0.42 | 0.673 | 1.000 |
|  | RT084-LPS | 3.17 | 4.35 | 0.73 | 0.467 | 1.000 |
|  | RT001-RT126 | 8.17 | 4.35 | 1.88 | 0.060 | 0.907 |
|  | RT001-700057 | -1.00 | 4.35 | -0.23 | 0.818 | 1.000 |
|  | RT001-LPS | 9.50 | 4.35 | 2.18 | 0.029 | 0.434 |
|  | RT126-LPS | 1.33 | 4.35 | 0.31 | 0.759 | 1.000 |
|  | Un-700057 | -5.00 | 4.35 | -1.15 | 0.250 | 1.000 |
|  | Un-LPS | -13.50 | 4.35 | -3.10 | 0.002 | 0.029 |
|  | 700057-LPS | 8.50 | 4.35 | 1.95 | 0.051 | 0.760 |
|  | Un-RT084 | -10.33 | 4.35 | -2.38 | 0.018 | 0.263 |
|  | Un-RT001 | -4.00 | 4.35 | -0.92 | 0.358 | 1.000 |
|  | Un-RT126 | -12.17 | 4.35 | -2.80 | 0.005 | 0.077 |
|  | 700057-RT084 | 5.33 | 4.35 | 1.23 | 0.220 | 1.000 |
|  | 700057-RT126 | 7.17 | 4.35 | 1.65 | 0.099 | 1.000 |
|  | RT001-RT084 | -6.33 | 4.35 | -1.46 | 0.145 | 1.000 |
| **24 h** | RT084-RT001 | 2.67 | 4.35 | 0.61 | 0.540 | 1.000 |
|  | RT084-RT126 | 9.83 | 4.35 | 2.26 | 0.024 | 0.359 |
|  | RT084-700057 | -1.33 | 4.35 | -0.31 | 0.759 | 1.000 |
|  | RT084-LPS | 7.83 | 4.35 | 1.80 | 0.072 | 1.000 |
|  | RT001-RT126 | 7.17 | 4.35 | 1.65 | 0.100 | 1.000 |
|  | RT001-LPS | 5.17 | 4.35 | 1.19 | 0.235 | 1.000 |
|  | Un-700057 | -6.00 | 4.35 | -1.38 | 0.168 | 1.000 |
|  | Un-LPS | -12.50 | 4.35 | -2.87 | 0.004 | 0.061 |
|  | 700057-LPS | 6.50 | 4.35 | 1.49 | 0.136 | 1.000 |
|  | Un-RT084 | -4.67 | 4.35 | -1.07 | 0.284 | 1.000 |
|  | Un-RT001 | -7.33 | 4.35 | -1.68 | 0.092 | 1.000 |
|  | Un-RT126 | -14.50 | 4.35 | -3.33 | 0.001 | 0.013 |
|  | 700057-RT001 | 1.33 | 4.35 | 0.31 | 0.759 | 1.000 |
|  | 700057-RT126 | 8.50 | 4.35 | 1.95 | 0.051 | 0.764 |
|  | LPS-RT126 | -2.00 | 4.35 | -0.46 | 0.646 | 1.000 |

**(M)**

| **Hour** | **Contrast** | **Test Statistic** | **Std. Error** | **Std. Test Statistic** | ***P*-value** | **Adj *P*-value^a^** |
| --- | --- | --- | --- | --- | --- | --- |
| **4 h** | Un-LPS | -5.333 | 4.354 | -1.225 | 0.221 | 1.000 |
|  | Un-RT126 | -5.500 | 4.354 | -1.263 | 0.207 | 1.000 |
|  | Un-RT001 | -7.167 | 4.354 | -1.646 | 0.100 | 1.000 |
|  | Un-RT084 | -13.000 | 4.354 | -2.985 | 0.003 | 0.042 |
|  | Un-700057 | -14.000 | 4.354 | -3.215 | 0.001 | 0.020 |
|  | LPS-RT126 | -0.167 | 4.354 | -0.038 | 0.969 | 1.000 |
|  | LPS-RT001 | -1.833 | 4.354 | -0.421 | 0.674 | 1.000 |
|  | LPS-RT084 | -7.667 | 4.354 | -1.761 | 0.078 | 1.000 |
|  | LPS-700057 | -8.667 | 4.354 | -1.990 | 0.047 | 0.698 |
|  | RT126-RT001 | -1.667 | 4.354 | -0.383 | 0.702 | 1.000 |
|  | RT126-RT084 | -7.500 | 4.354 | -1.722 | 0.085 | 1.000 |
|  | RT126-700057 | -8.500 | 4.354 | -1.952 | 0.051 | 0.764 |
|  | RT001-RT084 | -5.833 | 4.354 | -1.340 | 0.180 | 1.000 |
|  | RT001-700057 | -6.833 | 4.354 | -1.569 | 0.117 | 1.000 |
|  | RT084-700057 | -1.000 | 4.354 | -0.230 | 0.818 | 1.000 |
| **8 h** | Un-LPS | -10.500 | 4.343 | -2.418 | 0.016 | 0.234 |
|  | Un-RT126 | -5.500 | 4.343 | -1.266 | 0.205 | 1.000 |
|  | Un-RT001 | -11.333 | 4.343 | -2.609 | 0.009 | 0.136 |
|  | Un-RT084 | -14.000 | 4.343 | -3.223 | 0.001 | 0.019 |
|  | Un-700057 | -3.667 | 4.343 | -0.844 | 0.399 | 1.000 |
|  | LPS-RT001 | -0.833 | 4.343 | -0.192 | 0.848 | 1.000 |
|  | LPS-RT084 | -3.500 | 4.343 | -0.806 | 0.420 | 1.000 |
|  | RT126-RT001 | -5.833 | 4.343 | -1.343 | 0.179 | 1.000 |
|  | RT126-RT084 | -8.500 | 4.343 | -1.957 | 0.050 | 0.755 |
|  | RT001-RT084 | -2.667 | 4.343 | -0.614 | 0.539 | 1.000 |
|  | 700057-RT126 | 1.833 | 4.343 | 0.422 | 0.673 | 1.000 |
|  | 700057-LPS | 6.833 | 4.343 | 1.573 | 0.116 | 1.000 |
|  | 700057-RT001 | 7.667 | 4.343 | 1.765 | 0.078 | 1.000 |
|  | 700057-RT084 | 10.333 | 4.343 | 2.379 | 0.017 | 0.260 |
|  | RT126-LPS | 5.000 | 4.343 | 1.151 | 0.250 | 1.000 |
| **12 h** | Un-LPS | -10.667 | 4.357 | -2.448 | 0.014 | 0.215 |
|  | Un-RT126 | -12.667 | 4.357 | -2.907 | 0.004 | 0.055 |
|  | Un-RT001 | -4.500 | 4.357 | -1.033 | 0.302 | 1.000 |
|  | Un-RT084 | -12.667 | 4.357 | -2.907 | 0.004 | 0.055 |
|  | Un-700057 | -4.500 | 4.357 | -1.033 | 0.302 | 1.000 |
|  | LPS-RT126 | -2.000 | 4.357 | -0.459 | 0.646 | 1.000 |
|  | LPS-RT084 | -2.000 | 4.357 | -0.459 | 0.646 | 1.000 |
|  | RT126-RT084 | 0.000 | 4.357 | 0.000 | 1.000 | 1.000 |
|  | RT001-RT084 | -8.167 | 4.357 | -1.875 | 0.061 | 0.913 |
|  | RT001-700057 | 0.000 | 4.357 | 0.000 | 1.000 | 1.000 |
|  | 700057-RT126 | 8.167 | 4.357 | 1.875 | 0.061 | 0.913 |
|  | 700057-LPS | 6.167 | 4.357 | 1.415 | 0.157 | 1.000 |
|  | 700057-RT084 | 8.167 | 4.357 | 1.875 | 0.061 | 0.913 |
|  | RT001-LPS | 6.167 | 4.357 | 1.415 | 0.157 | 1.000 |
|  | RT001-RT126 | 8.167 | 4.357 | 1.875 | 0.061 | 0.913 |
| **24 h** | Un-LPS | -12.000 | 4.357 | -2.754 | 0.006 | 0.088 |
|  | Un-RT126 | -9.000 | 4.357 | -2.066 | 0.039 | 0.583 |
|  | Un-RT001 | -2.833 | 4.357 | -0.650 | 0.515 | 1.000 |
|  | Un-700057 | -5.000 | 4.357 | -1.148 | 0.251 | 1.000 |
|  | RT001-700057 | -2.167 | 4.357 | -0.497 | 0.619 | 1.000 |
|  | RT084-700057 | -6.833 | 4.357 | -1.568 | 0.117 | 1.000 |
|  | 700057-RT126 | 4.000 | 4.357 | 0.918 | 0.359 | 1.000 |
|  | 700057-LPS | 7.000 | 4.357 | 1.607 | 0.108 | 1.000 |
|  | RT126-LPS | 3.000 | 4.357 | 0.689 | 0.491 | 1.000 |
|  | RT001-LPS | 9.167 | 4.357 | 2.104 | 0.035 | 0.531 |
|  | RT001-RT126 | 6.167 | 4.357 | 1.415 | 0.157 | 1.000 |
|  | RT084-Un | 1.833 | 4.357 | 0.421 | 0.674 | 1.000 |
|  | RT084-RT001 | 4.667 | 4.357 | 1.071 | 0.284 | 1.000 |
|  | RT084-RT126 | 10.833 | 4.357 | 2.487 | 0.013 | 0.193 |
|  |  |  |  |  |  |  |

Each row tests the null hypothesis that the Sample 1 and Sample 2 distributions are the same. Asymptotic significances (2-sided tests) are displayed.

The significance level is considered less than 0.05. RT, ribotypes.

a. Significance values have been adjusted by the Bonferroni correction for multiple tests.
