# Supplementary material for: Surface layer protein A from hypervirulent Clostridioides difficile ribotypes induce significant changes in the gene expression of tight junctions and inflammatory response in human intestinal epithelial cells
Source: BMC Microbiol. 2022 Oct 27;22:259. doi: 10.1186/s12866-022-02665-0 (PMC9608920; doi:10.1186/s12866-022-02665-0)
Supplement: Supplementary file 6 — Supplementary Material 6 [file 12866_2022_2665_MOESM6_ESM.docx]

**Table S3** The ELISA results of inflammatory cytokines including (A) TNF-α, (B) IL-8 and (C) IL-1β in HT-29 cells upon treatment with SlpA (20 µg/mL) from *C. difficile* (RT126, RT001, RT084) and *C. difficile* ATCC 700057 at different time points (4, 8, 12 and 24 h).

**(A)**

| **Hour** | **Group 1** | **Group 2** | **Statistic** | ***P*-value** | **Adj *P*-value** | **Adj *P*-value significance** |
| --- | --- | --- | --- | --- | --- | --- |
| **4 h** | Un | LPS | 1.685865 | 0.091822 | 1 | ns |
| **4 h** | Un | RT126 | -0.30652 | 0.759208 | 1 | ns |
| **4 h** | Un | RT001 | 1.455975 | 0.1454 | 1 | ns |
| **4 h** | Un | RT084 | 2.605428 | 0.009176 | 0.550557 | ns |
| **4 h** | Un | 700057 | -0.15326 | 0.878193 | 1 | ns |
| **4 h** | LPS | RT126 | -1.99239 | 0.046329 | 1 | ns |
| **4 h** | LPS | RT001 | -0.22989 | 0.818177 | 1 | ns |
| **4 h** | LPS | RT084 | 0.919563 | 0.357801 | 1 | ns |
| **4 h** | LPS | 700057 | -1.83913 | 0.065897 | 1 | ns |
| **4 h** | RT126 | RT001 | 1.762495 | 0.077986 | 1 | ns |
| **4 h** | RT126 | RT084 | 2.911949 | 0.003592 | 0.215509 | ns |
| **4 h** | RT126 | 700057 | 0.15326 | 0.878193 | 1 | ns |
| **4 h** | RT001 | RT084 | 1.149454 | 0.250369 | 1 | ns |
| **4 h** | RT001 | 700057 | -1.60924 | 0.107565 | 1 | ns |
| **4 h** | RT084 | 700057 | -2.75869 | 0.005803 | 0.348203 | ns |
| **8 h** | Un | LPS | 0.688247 | 0.491297 | 1 | ns |
| **8 h** | Un | RT126 | 2.064742 | 0.038947 | 1 | ns |
| **8 h** | Un | RT001 | 3.441236 | 0.000579 | 0.034744 | ** |
| **8 h** | Un | RT084 | 2.752989 | 0.005905 | 0.354323 | ns |
| **8 h** | Un | 700057 | 1.376494 | 0.168669 | 1 | ns |
| **8 h** | LPS | RT126 | 1.376494 | 0.168669 | 1 | ns |
| **8 h** | LPS | RT001 | 2.752989 | 0.005905 | 0.354323 | ns |
| **8 h** | LPS | RT084 | 2.064742 | 0.038947 | 1 | ns |
| **8 h** | LPS | 700057 | 0.688247 | 0.491297 | 1 | ns |
| **8 h** | RT126 | RT001 | 1.376494 | 0.168669 | 1 | ns |
| **8 h** | RT126 | RT084 | 0.688247 | 0.491297 | 1 | ns |
| **8 h** | RT126 | 700057 | -0.68825 | 0.491297 | 1 | ns |
| **8 h** | RT001 | RT084 | -0.68825 | 0.491297 | 1 | ns |
| **8 h** | RT001 | 700057 | -2.06474 | 0.038947 | 1 | ns |
| **8 h** | RT084 | 700057 | -1.37649 | 0.168669 | 1 | ns |
| **12 h** | Un | LPS | 1.758854 | 0.078602 | 1 | ns |
| **12 h** | Un | RT126 | 3.441236 | 0.000579 | 0.034744 | ** |
| **12 h** | Un | RT001 | 1.682382 | 0.092495 | 1 | ns |
| **12 h** | Un | RT084 | 2.752989 | 0.005905 | 0.354323 | ns |
| **12 h** | Un | 700057 | 0.688247 | 0.491297 | 1 | ns |
| **12 h** | LPS | RT126 | 1.682382 | 0.092495 | 1 | ns |
| **12 h** | LPS | RT001 | -0.07647 | 0.939044 | 1 | ns |
| **12 h** | LPS | RT084 | 0.994135 | 0.320157 | 1 | ns |
| **12 h** | LPS | 700057 | -1.07061 | 0.284346 | 1 | ns |
| **12 h** | RT126 | RT001 | -1.75885 | 0.078602 | 1 | ns |
| **12 h** | RT126 | RT084 | -0.68825 | 0.491297 | 1 | ns |
| **12 h** | RT126 | 700057 | -2.75299 | 0.005905 | 0.354323 | ns |
| **12 h** | RT001 | RT084 | 1.070607 | 0.284346 | 1 | ns |
| **12 h** | RT001 | 700057 | -0.99413 | 0.320157 | 1 | ns |
| **12 h** | RT084 | 700057 | -2.06474 | 0.038947 | 1 | ns |
| **24 h** | Un | LPS | 3.441236 | 0.000579 | 0.034744 | ** |
| **24 h** | Un | RT126 | 2.752989 | 0.005905 | 0.354323 | ns |
| **24 h** | Un | RT001 | 2.064742 | 0.038947 | 1 | ns |
| **24 h** | Un | RT084 | 0.688247 | 0.491297 | 1 | ns |
| **24 h** | Un | 700057 | 1.376494 | 0.168669 | 1 | ns |
| **24 h** | LPS | RT126 | -0.68825 | 0.491297 | 1 | ns |
| **24 h** | LPS | RT001 | -1.37649 | 0.168669 | 1 | ns |
| **24 h** | LPS | RT084 | -2.75299 | 0.005905 | 0.354323 | ns |
| **24 h** | LPS | 700057 | -2.06474 | 0.038947 | 1 | ns |
| **24 h** | RT126 | RT001 | -0.68825 | 0.491297 | 1 | ns |
| **24 h** | RT126 | RT084 | -2.06474 | 0.038947 | 1 | ns |
| **24 h** | RT126 | 700057 | -1.37649 | 0.168669 | 1 | ns |
| **24 h** | RT001 | RT084 | -1.37649 | 0.168669 | 1 | ns |
| **24 h** | RT001 | 700057 | -0.68825 | 0.491297 | 1 | ns |
| **24 h** | RT084 | 700057 | 0.688247 | 0.491297 | 1 | ns |

**(B)**

| **Hour** | **Group 1** | **Group 2** | **Statistic** | ***P*-value** | **Adj *P*-value** | **Adj *P*-value significance** |
| --- | --- | --- | --- | --- | --- | --- |
| **4 h** | Un | LPS | 3.441236 | 0.000579 | 0.034744 | ** |
| **4 h** | Un | RT126 | 2.752989 | 0.005905 | 0.354323 | ns |
| **4 h** | Un | RT001 | 0.688247 | 0.491297 | 1 | ns |
| **4 h** | Un | RT084 | 1.529438 | 0.126156 | 1 | ns |
| **4 h** | Un | 700057 | 1.911798 | 0.055902 | 1 | ns |
| **4 h** | LPS | RT126 | -0.68825 | 0.491297 | 1 | ns |
| **4 h** | LPS | RT001 | -2.75299 | 0.005905 | 0.354323 | ns |
| **4 h** | LPS | RT084 | -1.9118 | 0.055902 | 1 | ns |
| **4 h** | LPS | 700057 | -1.52944 | 0.126156 | 1 | ns |
| **4 h** | RT126 | RT001 | -2.06474 | 0.038947 | 1 | ns |
| **4 h** | RT126 | RT084 | -1.22355 | 0.221122 | 1 | ns |
| **4 h** | RT126 | 700057 | -0.84119 | 0.400241 | 1 | ns |
| **4 h** | RT001 | RT084 | 0.841191 | 0.400241 | 1 | ns |
| **4 h** | RT001 | 700057 | 1.223551 | 0.221122 | 1 | ns |
| **4 h** | RT084 | 700057 | 0.38236 | 0.702195 | 1 | ns |
| **8 h** | Un | LPS | 2.752989 | 0.005905 | 0.354323 | ns |
| **8 h** | Un | RT126 | 2.064742 | 0.038947 | 1 | ns |
| **8 h** | Un | RT001 | 1.376494 | 0.168669 | 1 | ns |
| **8 h** | Un | RT084 | 3.441236 | 0.000579 | 0.034744 | ** |
| **8 h** | Un | 700057 | 0.688247 | 0.491297 | 1 | ns |
| **8 h** | LPS | RT126 | -0.68825 | 0.491297 | 1 | ns |
| **8 h** | LPS | RT001 | -1.37649 | 0.168669 | 1 | ns |
| **8 h** | LPS | RT084 | 0.688247 | 0.491297 | 1 | ns |
| **8 h** | LPS | 700057 | -2.06474 | 0.038947 | 1 | ns |
| **8 h** | RT126 | RT001 | -0.68825 | 0.491297 | 1 | ns |
| **8 h** | RT126 | RT084 | 1.376494 | 0.168669 | 1 | ns |
| **8 h** | RT126 | 700057 | -1.37649 | 0.168669 | 1 | ns |
| **8 h** | RT001 | RT084 | 2.064742 | 0.038947 | 1 | ns |
| **8 h** | RT001 | 700057 | -0.68825 | 0.491297 | 1 | ns |
| **8 h** | RT084 | 700057 | -2.75299 | 0.005905 | 0.354323 | ns |
| **12 h** | Un | LPS | 2.447101 | 0.014401 | 0.864062 | ns |
| **12 h** | Un | RT126 | 3.441236 | 0.000579 | 0.034744 | ** |
| **12 h** | Un | RT001 | 1.376494 | 0.168669 | 1 | ns |
| **12 h** | Un | RT084 | 2.370629 | 0.017758 | 1 | ns |
| **12 h** | Un | 700057 | 0.688247 | 0.491297 | 1 | ns |
| **12 h** | LPS | RT126 | 0.994135 | 0.320157 | 1 | ns |
| **12 h** | LPS | RT001 | -1.07061 | 0.284346 | 1 | ns |
| **12 h** | LPS | RT084 | -0.07647 | 0.939044 | 1 | ns |
| **12 h** | LPS | 700057 | -1.75885 | 0.078602 | 1 | ns |
| **12 h** | RT126 | RT001 | -2.06474 | 0.038947 | 1 | ns |
| **12 h** | RT126 | RT084 | -1.07061 | 0.284346 | 1 | ns |
| **12 h** | RT126 | 700057 | -2.75299 | 0.005905 | 0.354323 | ns |
| **12 h** | RT001 | RT084 | 0.994135 | 0.320157 | 1 | ns |
| **12 h** | RT001 | 700057 | -0.68825 | 0.491297 | 1 | ns |
| **12 h** | RT084 | 700057 | -1.68238 | 0.092495 | 1 | ns |
| **24 h** | Un | LPS | 2.752989 | 0.005905 | 0.354323 | ns |
| **24 h** | Un | RT126 | 3.441236 | 0.000579 | 0.034744 | ** |
| **24 h** | Un | RT001 | 2.064742 | 0.038947 | 1 | ns |
| **24 h** | Un | RT084 | 1.376494 | 0.168669 | 1 | ns |
| **24 h** | Un | 700057 | 0.688247 | 0.491297 | 1 | ns |
| **24 h** | LPS | RT126 | 0.688247 | 0.491297 | 1 | ns |
| **24 h** | LPS | RT001 | -0.68825 | 0.491297 | 1 | ns |
| **24 h** | LPS | RT084 | -1.37649 | 0.168669 | 1 | ns |
| **24 h** | LPS | 700057 | -2.06474 | 0.038947 | 1 | ns |
| **24 h** | RT126 | RT001 | -1.37649 | 0.168669 | 1 | ns |
| **24 h** | RT126 | RT084 | -2.06474 | 0.038947 | 1 | ns |
| **24 h** | RT126 | 700057 | -2.75299 | 0.005905 | 0.354323 | ns |
| **24 h** | RT001 | RT084 | -0.68825 | 0.491297 | 1 | ns |
| **24 h** | RT001 | 700057 | -1.37649 | 0.168669 | 1 | ns |
| **24 h** | RT084 | 700057 | -0.68825 | 0.491297 | 1 | ns |

**(C)**

| **Hour** | **Group 1** | **Group 2** | **Statistic** | ***p*-value** | **Adj *P*-value** | **Adj *P*-value significance** |
| --- | --- | --- | --- | --- | --- | --- |
| **4 h** | Un | LPS | 3.441236 | 0.000579 | 0.034744 | ** |
| **4 h** | Un | RT126 | 2.752989 | 0.005905 | 0.354323 | ns |
| **4 h** | Un | RT001 | 0.688247 | 0.491297 | 1 | ns |
| **4 h** | Un | RT084 | 1.529438 | 0.126156 | 1 | ns |
| **4 h** | Un | 700057 | 1.911798 | 0.055902 | 1 | ns |
| **4 h** | LPS | RT126 | -0.68825 | 0.491297 | 1 | ns |
| **4 h** | LPS | RT001 | -2.75299 | 0.005905 | 0.354323 | ns |
| **4 h** | LPS | RT084 | -1.9118 | 0.055902 | 1 | ns |
| **4 h** | LPS | 700057 | -1.52944 | 0.126156 | 1 | ns |
| **4 h** | RT126 | RT001 | -2.06474 | 0.038947 | 1 | ns |
| **4 h** | RT126 | RT084 | -1.22355 | 0.221122 | 1 | ns |
| **4 h** | RT126 | 700057 | -0.84119 | 0.400241 | 1 | ns |
| **4 h** | RT001 | RT084 | 0.841191 | 0.400241 | 1 | ns |
| **4 h** | RT001 | 700057 | 1.223551 | 0.221122 | 1 | ns |
| **4 h** | RT084 | 700057 | 0.38236 | 0.702195 | 1 | ns |
| **8 h** | Un | LPS | 2.752989 | 0.005905 | 0.354323 | ns |
| **8 h** | Un | RT126 | 2.064742 | 0.038947 | 1 | ns |
| **8 h** | Un | RT001 | 1.376494 | 0.168669 | 1 | ns |
| **8 h** | Un | RT084 | 3.441236 | 0.000579 | 0.034744 | ** |
| **8 h** | Un | 700057 | 0.688247 | 0.491297 | 1 | ns |
| **8 h** | LPS | RT126 | -0.68825 | 0.491297 | 1 | ns |
| **8 h** | LPS | RT001 | -1.37649 | 0.168669 | 1 | ns |
| **8 h** | LPS | RT084 | 0.688247 | 0.491297 | 1 | ns |
| **8 h** | LPS | 700057 | -2.06474 | 0.038947 | 1 | ns |
| **8 h** | RT126 | RT001 | -0.68825 | 0.491297 | 1 | ns |
| **8 h** | RT126 | RT084 | 1.376494 | 0.168669 | 1 | ns |
| **8 h** | RT126 | 700057 | -1.37649 | 0.168669 | 1 | ns |
| **8 h** | RT001 | RT084 | 2.064742 | 0.038947 | 1 | ns |
| **8 h** | RT001 | 700057 | -0.68825 | 0.491297 | 1 | ns |
| **8 h** | RT084 | 700057 | -2.75299 | 0.005905 | 0.354323 | ns |
| **12 h** | Un | LPS | 2.447101 | 0.014401 | 0.864062 | ns |
| **12 h** | Un | RT126 | 3.441236 | 0.000579 | 0.034744 | ** |
| **12 h** | Un | RT001 | 1.376494 | 0.168669 | 1 | ns |
| **12 h** | Un | RT084 | 2.370629 | 0.017758 | 1 | ns |
| **12 h** | Un | 700057 | 0.688247 | 0.491297 | 1 | ns |
| **12 h** | LPS | RT126 | 0.994135 | 0.320157 | 1 | ns |
| **12 h** | LPS | RT001 | -1.07061 | 0.284346 | 1 | ns |
| **12 h** | LPS | RT084 | -0.07647 | 0.939044 | 1 | ns |
| **12 h** | LPS | 700057 | -1.75885 | 0.078602 | 1 | ns |
| **12 h** | RT126 | RT001 | -2.06474 | 0.038947 | 1 | ns |
| **12 h** | RT126 | RT084 | -1.07061 | 0.284346 | 1 | ns |
| **12 h** | RT126 | 700057 | -2.75299 | 0.005905 | 0.354323 | ns |
| **12 h** | RT001 | RT084 | 0.994135 | 0.320157 | 1 | ns |
| **12 h** | RT001 | 700057 | -0.68825 | 0.491297 | 1 | ns |
| **12 h** | RT084 | 700057 | -1.68238 | 0.092495 | 1 | ns |
| **24 h** | Un | LPS | 2.752989 | 0.005905 | 0.354323 | ns |
| **24 h** | Un | RT126 | 3.441236 | 0.000579 | 0.034744 | ** |
| **24 h** | Un | RT001 | 2.064742 | 0.038947 | 1 | ns |
| **24 h** | Un | RT084 | 1.376494 | 0.168669 | 1 | ns |
| **24 h** | Un | 700057 | 0.688247 | 0.491297 | 1 | ns |
| **24 h** | LPS | RT126 | 0.688247 | 0.491297 | 1 | ns |
| **24 h** | LPS | RT001 | -0.68825 | 0.491297 | 1 | ns |
| **24 h** | LPS | RT084 | -1.37649 | 0.168669 | 1 | ns |
| **24 h** | LPS | 700057 | -2.06474 | 0.038947 | 1 | ns |
| **24 h** | RT126 | RT001 | -1.37649 | 0.168669 | 1 | ns |
| **24 h** | RT126 | RT084 | -2.06474 | 0.038947 | 1 | ns |
| **24 h** | RT126 | 700057 | -2.75299 | 0.005905 | 0.354323 | ns |
| **24 h** | RT001 | RT084 | -0.68825 | 0.491297 | 1 | ns |
| **24 h** | RT001 | 700057 | -1.37649 | 0.168669 | 1 | ns |
| **24 h** | RT084 | 700057 | -0.68825 | 0.491297 | 1 | ns |

RT, ribotype
